# Oxygen-Promoted Dehydrogenation on Size-Selected Cu–Pd Pentamer Clusters for Low-Temperature Hydrogen Release from Cyclic Hydrocarbons

**DOI:** 10.1021/acsnano.6c12121

**Published:** 2026-10-27

**Authors:** Mykhailo Vaidulych, Yonghyuk Lee, Federico Loi, Petr Vitek, Michael Vorochta, Stanislav Valtera, Ivan Khalakhan, Claudia de Melo, Samiran Chakraborty, Karolína Simkovičová, Yves Garreau, Benjamin Voisin, Bilal Bawab, Jan M. Macak, Muntaseer Bunian, Moon-Hyung Jang, Yu Lei, Philippe Sautet, Iva Matolinová, Alessandro Coati, Pascal Andreazza, Anastassia N. Alexandrova, Stefan Vajda

**Affiliations:** 1 Department of Nanocatalysis, Heyrovský Institute, Czech Academy of Sciences, Dolejškova 2155/3, 18200 Prague 8, Czech Republic; 2 Department of Chemistry and Biochemistry, 8783 University of California, Los Angeles, California 90095, United States; 3 Department of Chemical and Biochemical Engineering, Dongguk University, Seoul 04620, Republic of Korea; 4 Department of Surface and Plasma Science, Faculty of Mathematics and Physics, Charles University, VHolešovičkách 2, 18000 Prague, Czech Republic; 5 Université d’Orléans, CNRS, ICMN UMR7374, 1b Rue de la Férollerie, 45071 Orléans, France; 6 55536 Synchrotron SOLEIL, L’Orme des Merisiers, Départementale 128, 91190 Saint-Aubin, France; 7 Center of Materials and Nanotechnologies, Faculty of Chemical Technology, University of Pardubice, Pardubice 53002, Czech Republic; 8 Central European Institute of Technology, Brno University of Technology, Purkynova 123, 612 00 Brno, Czech Republic; 9 Department of Chemical and Materials Engineering, 14843 University of Alabama in Huntsville, 301 Sparkman Drive, Huntsville, Alabama 35899, United States

**Keywords:** cyclohexene, benzene, H_2_ production and storage, oxidative dehydrogenation (ODH), bimetallic CuPd clusters, NAP-XPS, in situ GIWAXS, DFT calculations

## Abstract

Despite extensive studies on cycloalkane and cycloalkene dehydrogenation for liquid organic hydrogen carrier applications, the mechanisms governing selective H_2_ release under mild conditions remain poorly understood. Here, subnanometer Cu_*x*_Pd_*y*_ pentamer clusters supported on ZrO_2_ represent a highly active and selective class of low-temperature dehydrogenation catalysts. Pd_5_ exhibits the highest benzene and molecular H_2_ formation rates from dehydrogenation of cyclohexene, followed by Cu_2_Pd_3_ and Cu_3_Pd_2_, whereas Cu_5_ is inactive. All active catalysts favored H_2_ over H_2_O as the hydrogen-containing product and completely suppressed combustion pathways, with no detectable CO_2_ formation. Oxygen-lean conditions further favored H_2_ formation across all active catalysts, with H_2_ selectivity exceeding 99% for Pd_5_. Kinetic analysis of Cu_3_Pd_2_ reveals positive fractional apparent orders in both C_6_H_10_ and O_2_ and an effective apparent activation energy of approximately 66 kJ·mol^–1^, while reactant composition and temperature systematically control H_2_/H_2_O branching. *In situ* XPS and *in situ* GIWAXS reveal that catalytic activity and cluster stability arise from the interplay of atomic composition, electronic structure, metal–oxygen interactions, and the chemical environment, with Cu exhibiting a composition-sensitive redox response and Pd-containing clusters accommodating oxygen while retaining electronically active Pd-derived states near the Fermi level. DFT/MLIP free-energy calculations identify kinetically accessible O-assisted first C–H activation pathways involving H transfer to cluster-bound O, with barriers strongly dependent on cluster composition and O configuration. These findings establish an ensemble-based structure–activity framework for controlling dehydrogenation pathways and provide design principles for catalysts that efficiently promote selective hydrogen release suitable for hydrogen carrier applications.

## Graphical abstract

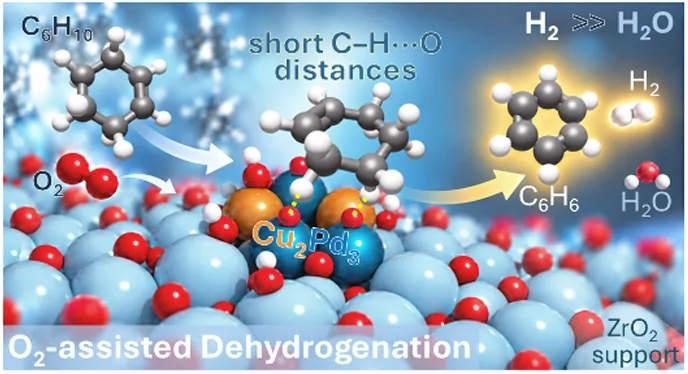

## 1 Introduction

The transition toward a hydrogen-based green energy economy requires efficient methods for hydrogen storage and release, as hydrogen’s low volumetric density makes its direct handling technologically demanding.^1^ Liquid organic hydrogen carriers (LOHCs) provide a promising route because they enable hydrogen to be stored in a stable liquid form and release it on demand through catalytic dehydrogenation (DH).^2–5^ Cycloalkanes (such as cyclohexane, methylcyclohexane, perhydro­benzyltoluene, decalin, and perhydro-*N*-ethylcarbazole) represent an attractive class of LOHCs due to their high hydrogen density, liquid phase under ambient conditions, and compatibility with existing fuel infrastructure.^5–7^ However, dehydrogenation is inherently endothermic and requires substantial energy input to overcome C–H bond activation barriers, resulting in high operating temperatures that limit the practical implementation of LOHC technologies.^8^

Early studies of cyclohexane dehydrogenation under oxygen-free conditions established that the reaction proceeds via di-σ-bonded adsorption followed by stepwise hydrogen elimination to cyclohexene and cyclohexadiene, ultimately yielding benzene. In this pathway, the π → σ (pi-to-sigma) reorientation of the cyclohexene intermediate constitutes a major kinetic bottleneck.^9–13^ Because the transformation of cycloalkanes to their corresponding aromatic products generally proceeds through alkene intermediates, cycloalkenes represent a mechanistically relevant node common to the dehydrogenation pathways of cyclohexane and related LOHC systems.^7,13–15^ Accordingly, cyclohexene was selected as a model probe molecule to provide direct insight into C–H bond activation and adsorption geometry, enabling decoupling of the initial di-σ-bond activation from subsequent dehydrogenation and/or oxidation steps while offering a sensitive measure of the catalyst’s ability to control reaction kinetics and selectivity.

Classical adsorption studies on model metal surfaces show that cyclohexene dehydrogenation is both kinetically limited and thermodynamically unfavorable at low temperatures, requiring substantial activation to proceed efficiently.^11,12^ As a result, purely thermal dehydrogenation typically demands high temperatures where catalyst deactivation and carbon deposition (coking) become problematic.^16,17^ On the other hand, oxidative dehydrogenation (ODH) offers an attractive alternative pathway in which adsorbed oxygen fundamentally reshapes reaction energetics, enabling olefin or aromatic formation at substantially lower temperatures.^18–21^ In this regime, oxygen acts not only as a hydrogen acceptor but also as an electronic promoter, withdrawing electron density from metal sites (Pt, Pd, Ni, Co, etc.)^22–26^ and thereby lowering the barrier for C–H cleavage. In contrast to nonoxidative DH, where hydrogen recombines and desorbs as H_2_, ODH removes hydrogen via reaction with surface oxygen to form water, reducing the thermodynamic and kinetic constraints associated with H–H recombination. The exothermic formation of water further mitigates catalyst deactivation by suppressing coke formation and accumulation through oxidative removal of carbonaceous surface residues. As a result, ODH is often more energy-efficient and can deliver high benzene yields at lower temperatures than thermal DH. However, at higher oxygen coverages and elevated temperatures, oxidative pathways increasingly shift toward deep oxidation, promoting C–C bond cleavage and combustion to CO_2_, thereby reducing selectivity.^17,20^ While oxidative dehydrogenation offers a lower-temperature route to unsaturated hydrocarbons, its intrinsic consumption of hydrogen prevents direct application in LOHC systems, where efficient recovery of molecular H_2_ is essential. Consequently, developing catalysts that enable low-temperature dehydrogenation while preserving H_2_ release remains a central challenge.

Recent work has suggested that the balance between oxidative and nonoxidative pathways in alkene conversion is more complex than the classical ODH picture implies. In our previous study on nanostructured Pd and Pd–Pt films supported on titania, we observed substantial formation of free hydrogen even under nominal ODH conditions, indicating that a dehydrogenation-like pathway must coexist with the oxidative route.^20^ Notably, these observations are consistent with earlier nonoxidative DH study by Somorjai et al., who demonstrated that small amounts of strongly adsorbed surface oxygen can withdraw electron density from Pt surface atoms and thus promote C–H cleavage at undercoordinated kinked sites.^23^ At the same time, it has been shown that high oxygen coverage suppresses dehydrogenation, when oxygen saturates surface sites or diffuses subsurface, shifting reactivity toward complete inhibition of hydrocarbon conversion to benzene.^19,23^ Oxygen therefore exerts a coverage-dependent effect, acting as a promoter at low coverage and an inhibitor at high coverage.

Despite these advances, the way oxygen governs the balance between DH-like hydrogen release and classical ODH pathways remains poorly understood. While it is widely recognized that the oxidation state of metal sites influences activity and selectivity of conventional catalysts, far less is known about how oxygen interacts with fluxional catalytic ensembles to determine whether hydrogen is removed as H_2_ or H_2_O, how it modifies the mechanism of C–H activation, and how these effects influence the metal electronic structure and its interaction with the support.

Recent developments in catalyst design suggest that subnanometer, size-selected clusters offer a unique opportunity to explore these questions.^17,27–29^ Unlike extended surfaces or conventional nanoparticles, such clusters exhibit pronounced quantum size effects, strong structural fluxionality, and highly tunable electronic properties. At the same time, the multitude of accessible geometries and electronic states introduces substantial complexity,^30–33^ making it particularly compelling to understand how oxygen-induced electronic effects interplay with cluster-specific catalytic behaviors. Prior theoretical and experimental studies on cobalt clusters, for example, have shown that oxidation of small clusters weakens cyclohexane binding and enhances low-temperature activity, demonstrating that the oxidation state of ultrasmall clusters can be a decisive factor in dehydrogenation kinetics, while the reaction feed composition can strongly influence selectivity, with oxygen-lean conditions effectively suppressing CO_2_ formation.^34^ Bimetallic systems offer additional control, as synergistic interactions between different metal atoms enable atom-by-atom tuning of electronic structure, adsorption strength, and reaction pathways. In particular, CuPd clusters have emerged as promising candidates for selective dehydrogenation, where the catalytic performance can be tailored through precise control of size and composition.^35^

In this work, size-selected Cu_5_, Pd_5_, and bimetallic Cu_*x*_Pd_*y*_ pentamer clusters (*x* + *y* = 5) were produced with atomic precision in both size and composition, supported on an ultrathin ZrO_2_ film (see schematic representation in Scheme 1a). All active catalysts showed exceptional performance with high activity and 100% selectivity toward benzene formation, demonstrating complete suppression of combustion channels with no detectable CO_2_ formation and, to the best of our knowledge, significantly outperforming previously reported cluster-based catalysts, including pure Pd clusters.^35,36^ Using temperature-programmed reaction (TPR) under atmospheric conditions, combined with near ambient pressure X-ray photoelectron spectroscopy (NAP-XPS), *in situ* grazing-incidence wide-angle X-ray scattering (GIWAXS), and theoretical approaches using density functional theory (DFT) and machine learning (ML), we elucidate how catalytic properties evolve with cluster composition and how oxygen tunes the reaction pathways during cyclohexene conversion, through a combined contribution of both chemical and morphological features. The Cu–Pd synergy enables efficient utilization of both metals, delivering exceptional catalytic performance and composition-dependent stability, as evidenced by TPR and *in situ* GIWAXS. The *in situ* spectroscopy reveals composition-dependent redox responses of Cu and Pd under the reactive environment, whereas theoretical analysis shows that oxygenated CuPd and Pd ensembles retain electronically active Pd-derived states while forming local O/OH motifs that stabilize cyclohexene adsorption geometries relevant to C–H activation without excessive binding. A minimum oxygen concentration is therefore required to initiate dehydrogenation under mild conditions, whereas an oxygen deficiency increasingly directs hydrogen toward H_2_ formation and effectively suppresses H_2_O production.

**Scheme 1:**
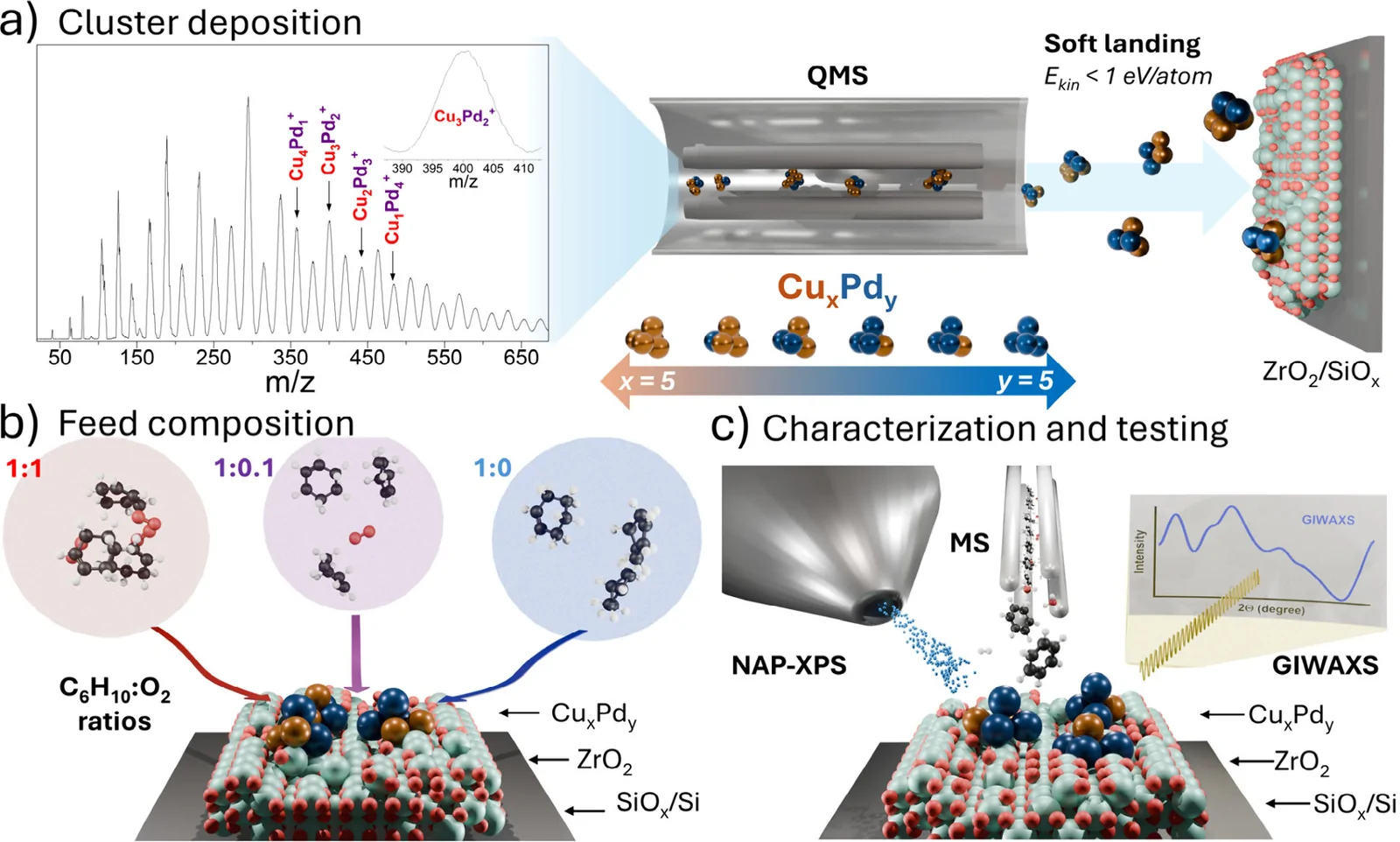
Representation of the Experimental Workflow^a^

a (a) Cluster synthesis in the gas phase using a molecular beam source and a representative mass spectrum obtained for bimetallic clusters. Unmarked peaks in the mass spectrum correspond to cluster compositions not used in this study. 10% of atomic monolayer equivalent (%ML) deposited on the surface of ZrO_2_ support for all cluster compositions. (b) Catalytic conditions used during TPR testing. (c) *In situ* characterization by TPR, NAP-XPS, and GIWAXS under technique-specific pressure conditions.

These findings extend beyond the oxidative dehydrogenation of cyclohexene and its relevance to LOHC systems, providing general insight into the dehydrogenation of cyclic hydrocarbons and highlighting the decisive role of substoichiometric oxygen-lean conditions in steering selectivity and reaction pathways, revealing how oxygen governs these processes for effective hydrocarbon transformations. Cyclohexene thereby serves as a mechanistically accessible model substrate for establishing oxygen-controlled catalytic principles that can be further explored by using saturated cyclic hydrocarbons and technologically relevant LOHC systems.

## 2 Results and Discussion

### 2.1 Catalytic Activity

The bimetallic Cu_*x*_Pd_*y*_ pentamers, as well as pure Cu_5_ and Pd_5_ clusters, supported on ZrO_2_ were prepared using a surfactant-free gas-phase synthesis method with mass selection, providing precise control over composition and coverage of the resulting catalysts (see Scheme 1). The catalytic performance of the supported clusters was investigated by using TPR combined with mass spectrometry (MS) under realistic reaction conditions of atmospheric pressure (1.1 bar) and mild temperatures. The averaged benzene and hydrogen formation rates are summarized in Figure 1a,b and are provided for three different C_6_H_10_:O_2_ reaction ratios: 1:1 (stoichiometric for ODH reaction), 1:0.1 (oxygen-lean ODH), and 1:0 (DH conditions with no O_2_). For the 1:1 feed, two consecutive cycles were performed, and only the second cycle is discussed below, as the first ramp is typically influenced by residual carbonaceous species, leading to their removal and to catalyst surface transformation or activation. For completeness, the time-resolved evolution of reactants and products across the full temperature profile is shown in Figure S1 in the Supporting Information.

**Figure 1:**
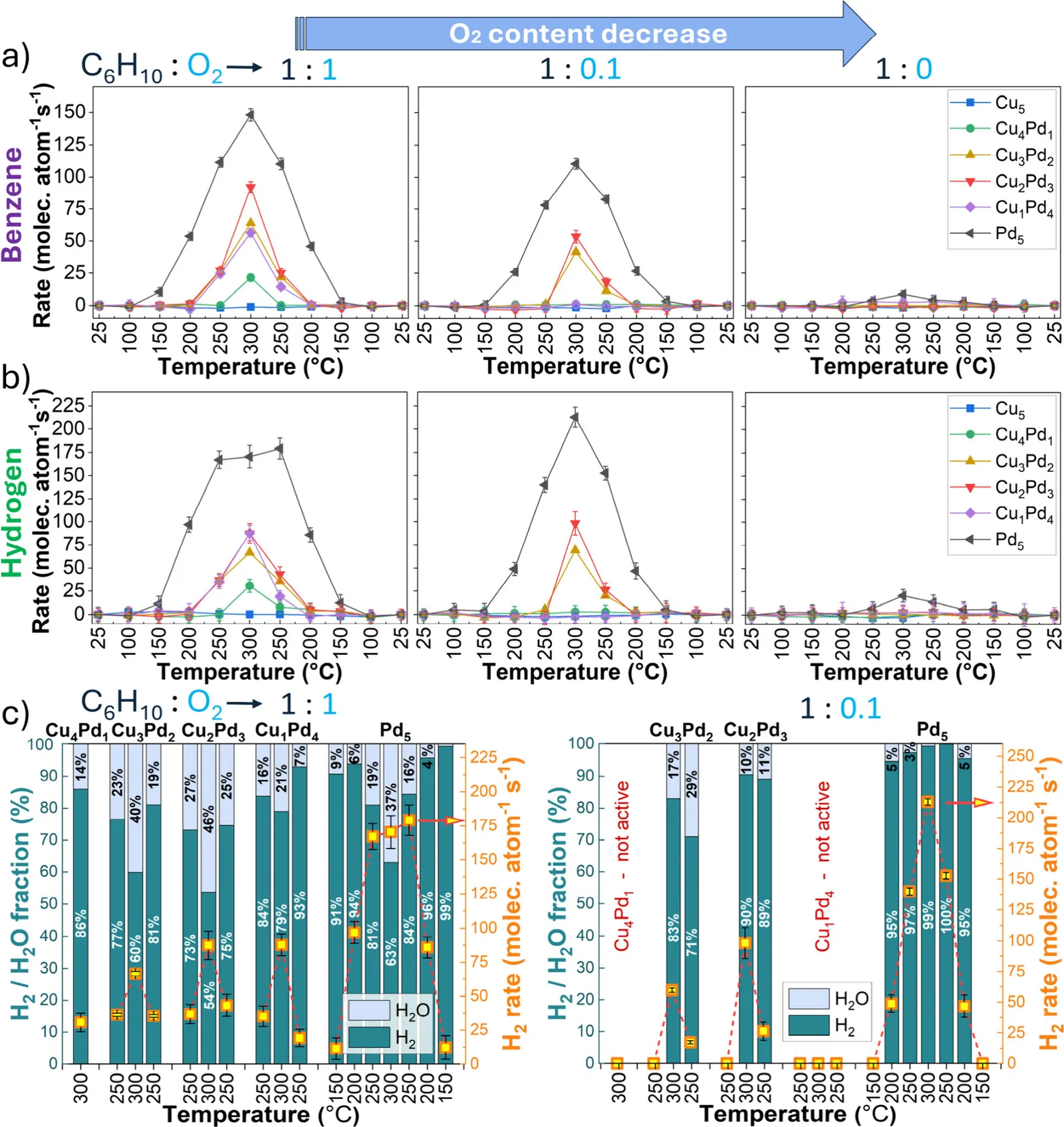
Averaged rates of benzene (a) and hydrogen (b) production as a function of temperature measured for the full series of cluster-based catalysts under different dehydrogenation conditions with C_6_H_10_:O_2_ ratios of 1:1, 1:0.1, and 1:0, respectively; (c) H_2_/H_2_O production fractions for all active catalysts obtained under 1:1 and 1:0.1 conditions, together with the corresponding H_2_ production rates (orange line). The reported catalytic rates correspond to the second temperature ramp, after the initial catalyst conditioning, and were averaged over the stable portion of each isothermal measurement.

In Figure 1a, under a 1:1 feed, Pd_5_ displayed the highest activity, becoming active at 150 °C with benzene formation rates of 11 molecules per catalyst atom per second (molec.·atom^–1^·s^–1^; for simplicity, we will refer to rates as s^–1^ throughout the text.). The rate increased steadily with temperature, reaching 148 s^–1^ at 300 °C, with a nearly identical profile during the cooling ramp. In contrast, Cu_5_ was completely inactive across the entire temperature range, whereas all bimetallic clusters exhibited a nonmonotonic, composition-dependent activity. The most active bimetallic cluster was Cu_2_Pd_3_, followed by Cu_3_Pd_2_ and Cu_1_Pd_4_, with benzene formation rates at 300 °C of 92, 64, and 56 s^–1^, respectively. All three compositions were already active at 250 °C, producing 25 s^–1^ of C_6_H_6_. For Cu_4_Pd_1_, the benzene rate at 300 °C was 22 s^–1^, the lowest among the bimetallic compositions yet still more than 5-fold and 2-fold higher than the activity reported for pure Pd_2_ and Pd_4_ clusters on ZrO_2_ under comparable conditions, respectively (while Pd_1_/ZrO_2_ showed no catalytic activity).^35^ Notably, previously studied CuPd tetramers on ZrO_2_ or UNCD were inactive in this temperature range.^35^ All active catalysts exhibited 100% selectivity toward benzene, with no detectable CO_2_ formation, demonstrating complete suppression of combustion channels, significantly outperforming previously reported pure Pd clusters on UNCD support, where combustion of C_6_H_12_ to CO_2_ was significant.^36^

Under oxygen-deficient conditions (1:0.1 ratio), only Pd_5_, Cu_2_Pd_3_, and Cu_3_Pd_2_ remained active. Pd_5_ showed robust performance starting at 200 °C, albeit with approximately a 30–50% lower benzene rate compared to stoichiometric ODH conditions across the active temperature range, e.g., 110 vs 148 s^–1^ at 300 °C. Cu_2_Pd_3_ also exhibited reduced activity at 300 °C (53 vs 92 s^–1^) and during the 250 °C down-ramp (18 vs 25 s^–1^), while Cu_3_Pd_2_ showed a marginally lower but comparable activity. Importantly, in the complete absence of oxygen, none of the bimetallic catalysts showed measurable activity, while Pd_5_ exhibited a benzene production rate of 9 s^–1^ only at 300 °C.

Contrary to the expected predominance of water formation as the primary byproduct for the stoichiometric ODH reaction, all active catalysts produced substantial amounts of free H_2_ under both oxygen concentrations, as shown in Figure 1b. With a C_6_H_10_:O_2_ ratio of 1:1, Pd_5_ produced hydrogen with a rate up to 180 s^–1^ during the down-ramp at 250 °C, whereas under oxygen-deficient conditions (1:0.1), the H_2_ rate reached an even higher value of 213 s^–1^ at 300 °C, which, to the best of our knowledge, is an exceptional result for an ODH-like reaction. Similarly, all bimetallic clusters released hydrogen with composition-dependent rates. To rationalize this behavior, we introduce the oxygen-lean oxidative dehydrogenation, defined as a nominally ODH regime operated under oxygen-deficient conditions in which H_2_ release remains predominant over H_2_O formation.

Figure 1c illustrates the relative partitioning of hydrogen from C_6_H_10_ into either H_2_O (through oxidative removal) or free H_2_ (DH-like removal). At a 1:1 ratio, the water fraction increased with temperature for all catalysts, with Cu_2_Pd_3_ producing the highest fraction (46%) and Cu_4_Pd_1_ the lowest (14%) at the maximum temperature of 300 °C. This trend is consistent with the literature on oxidative dehydrogenation, showing that oxygen activation, a highly endothermic process,^25,37,38^ becomes increasingly efficient at elevated temperatures and generates reactive surface oxygen species that facilitate C–H abstraction and subsequent H_2_ uptake via water formation.^21,34,39^ Hence, we observe a higher H_2_ production ratio at lower temperatures. Notably, the rate of H_2_ formation generally increased with increasing Pd content in the clusters, a behavior that remained consistent across both oxygen ratios. Under O_2_-deficient conditions, the water fraction decreased dramatically, where Cu_2_Pd_3_ and Pd_5_ produced 90% (98 s^–1^) and 99% (213 s^–1^) of H_2_ at 300 °C, respectively. However, Pd_5_ clearly outperformed bimetallic Cu_2_Pd_3_ and Cu_3_Pd_2_ in the low-temperature regime, producing almost exclusively H_2_ (above 95%) with high rates at temperatures as low as 200 °C. The absence of catalytic activity for the bimetallic clusters in the absence of O_2_, together with the significantly lower activity of Pd_5_, underlines the essential role of oxygen in initiating C–H bond cleavage, as addressed later in the paper.

When normalizing benzene and hydrogen formation rates per Pd atom in the cluster (Figure S2), the apparent differences in activity between catalysts at the highest temperature of 300 °C diminished substantially. Pd_5_, Cu_2_Pd_3_, and Cu_3_Pd_2_ exhibited nearly identical Pd-normalized activities with benzene rate of 155 ± 7 molec.·Pd_atom_^–1^·s^–1^ for 1:1 gas ratio and 100 ± 10 molec.·Pd_atom_^–1^·s^–1^ for 1:0.1 ratio, respectively, whereas Cu_1_Pd_4_ and Cu_4_Pd_1_ had lower or no activity (for the 1:1 and 1:0.1 ratios, respectively, in Figure S2), which may, among other factors, reflect reduced thermal stability, as discussed in the next sections.

### 2.2 Kinetic Analysis of Cyclohexene Dehydrogenation

To quantify the kinetic dependence of benzene formation on the two reactants, Cu_3_Pd_2_ was selected as a representative highly active and stable bimetallic catalyst. For each selected C_6_H_10_ or O_2_ partial pressure, benzene formation rates were measured over a complete temperature series at 270, 280, 290, and 300 °C while the concentration of the second reactant, total pressure, and total gas flow were maintained constant. The corresponding reactant partial pressures and concentrations are given in section 4.3 and were used for the reaction-order analysis. The extended measurement sequence also enabled the effects of reactant partial pressure, temperature, and gradual changes in catalyst activity to be evaluated separately.

#### 2.2.1 Apparent Reaction Orders

The apparent reaction orders were determined from the slopes of the logarithmic dependence of the benzene formation rate on reactant partial pressure at constant temperature (Figure S3). Over the common 280–300 °C kinetic range, the directly measured C_6_H_10_ reaction orders were 0.59, 0.74, and 0.83 at 280, 290, and 300 °C, respectively. The corresponding O_2_ reaction orders were 0.64, 0.73, and 0.88. Thus, benzene formation exhibits positive fractional-order dependence on both cyclohexene and oxygen, with the apparent dependence on both reactants becoming stronger as the temperature is increased, consistent with coverage effects expected for a surface-mediated reaction, where the coverage of adsorbed reactants and reaction intermediates, and the balance between their formation and consumption, vary with reaction conditions.^19^ The 270 °C C_6_H_10_-pressure series exhibited a nonmonotonic rate response and was not adequately represented by a single power-law relationship. Quantitative comparison of the reaction orders was therefore restricted to the common 280–300 °C range. The mean directly measured apparent orders over this range were approximately 0.72 in C_6_H_10_ and 0.75 in O_2_. These values are regarded as condition-specific apparent reaction orders rather than universal intrinsic differential orders.

Because repeated standard-feed (0.3% C_6_H_10_:0.3% O_2_) measurements showed gradual catalyst deactivation during the extended kinetic sequence, a sensitivity analysis was performed by normalizing the measured rates to the standard-feed activity expected at the corresponding experimental time. This reference activity was obtained by interpolating between adjacent measurements under standard conditions. The resulting normalized C_6_H_10_ orders were 0.52, 0.68, and 0.75 at 280, 290, and 300 °C, respectively, whereas the corresponding normalized O_2_ orders were 0.56, 0.63, and 0.77. The mean normalized values were therefore approximately 0.65 and 0.66, respectively. The modest decrease relative to the directly measured values shows that catalyst evolution contributes some systematic uncertainty to the numerical slopes but does not change the principal conclusion that benzene formation is positive fractional order in both reactants.

The temperature dependence of the apparent orders further indicates that the catalytic rate cannot be described by a single composition- and coverage-independent power law over the full experimental range. Instead, changes in reactant and adsorbate coverage and/or in the population of reaction-relevant cluster states are likely to contribute to the measured kinetic response.

#### 2.2.2 Apparent Activation Energy (*E*_app_) and Feed-Composition Dependence

Arrhenius analysis of repeated temperature ramps under the standard feed yielded apparent activation energies in the range of approximately 61–70 kJ·mol^–1^, or 0.63–0.73 eV (see Figure S4a). Considering the repeated standard-feed measurements together gives an effective apparent activation energy of approximately 66 kJ·mol^–1^ (0.68 eV) for benzene formation over 270–300 °C.

This value represents the overall temperature dependence of benzene formation under the specified reaction conditions and should not be identified directly with the activation barrier of an individual elementary C–H-cleavage step. In a supported subnanometer-cluster catalyst, the measured *E*_app_ can contain contributions from reactant adsorption, surface coverage, change of the cluster oxidation state and structure, hydrogen removal, product desorption, and changes in the relative kinetic importance of individual elementary steps.

A pronounced feed-composition dependence of *E*_app_ was also observed (Figure S4b,c). At constant O_2_ partial pressure, decreasing the C_6_H_10_ concentration from 0.30 to 0.15% reduced the apparent activation energy from approximately 66 to 33 kJ·mol^–1^ (0.68 and 0.34 eV, respectively), although the absolute benzene formation rate decreased. The lower rate therefore reflects the reduced availability and coverage of cyclohexene rather than an increase in the apparent energetic barrier. In other words, a lower *E*_app_ signifies a weaker temperature dependence and does not imply a higher absolute reaction rate.

The opposite trend was observed when the oxygen partial pressure was varied (Figure S4c). At constant C_6_H_10_ concentration, decreasing O_2_ from 0.30 to 0.20% increased the apparent activation energy to approximately 84 kJ·mol^–1^ (0.87 eV). Together with the positive fractional O_2_ reaction order, this behavior indicates that oxygen availability affects the kinetically relevant catalytic state and/or the sequence of surface reactions. Oxygen may contribute to maintaining a catalytically active oxygenated cluster environment and to the removal of adsorbed hydrogen, although the present kinetic measurements cannot distinguish these possibilities at the elementary-step level. The composition-dependent activation energies are therefore interpreted as evidence for coverage- and catalyst-state-dependent kinetics rather than as changes in a single intrinsic transition-state barrier.

#### 2.2.3 Hydrogen-Product Branching between H_2_ and H_2_O

The pressure-dependent measurements also provide direct information on the partitioning of hydrogen removed during cyclohexene dehydrogenation (see Figure S4d–f). Increasing the C_6_H_10_ concentration systematically shifted the hydrogen-containing products toward H_2_, whereas increasing the O_2_ concentration shifted the distribution toward H_2_O. Temperature produced a similar change in branching: under the standard-feed conditions, the average fraction of removed hydrogen detected as H_2_ decreased from approximately 83.3% at 270 °C to 66.1% at 300 °C, that is corresponding well to the results presented in Figure 1c.

To examine this branching quantitatively, the benzene formation rate was partitioned according to the measured H_2_/H_2_O product distribution. Over 280–300 °C, the selectivity-weighted C_6_H_10_ order was approximately 0.97 for the H_2_-associated benzene rate but close to zero for the H_2_O-associated rate. In contrast, the corresponding apparent O_2_ orders were approximately 0.55 and 0.90, respectively. Thus, increasing hydrocarbon availability preferentially promotes the H_2_-associated branch, whereas increasing oxygen availability more strongly promotes the H_2_O-associated branch. These trends are consistent with competition between recombination of adsorbed hydrogen, and oxygen-assisted removal of surface hydrogen through O*/OH*-containing intermediates. The selectivity-weighted reaction orders should not be interpreted as reaction orders of two completely independent elementary pathways, because H_2_ and H_2_O formation may remain kinetically coupled. Secondary oxidation of initially formed H_2_, reversible hydrogen transfer, changes in O/H coverage, and changes in cluster oxidation state may also contribute to the observed branching.

Partitioning the benzene rate according to the H_2_/H_2_O distribution also gives strongly different apparent temperature dependences for the two constructed rates. Because these quantities contain the temperature dependence of both the overall benzene rate and the H_2_/H_2_O branching ratio, they are not interpreted here as elementary activation energies for H_2_- and H_2_O-forming pathways.

The extended kinetic sequence also provided an assessment of catalyst durability under repeated thermal and chemical perturbations. Following approximately 11 days of kinetic measurements involving repeated temperature ramps and changes in reactant composition, the same Cu_3_Pd_2_ sample was monitored for an additional 24 h under standard reaction conditions at 290 °C. Benzene production decreased by less than 3% over this period, while the H_2_ signal showed a slight increase (Figure S5), demonstrating substantial catalytic stability of Cu_3_Pd_2_ on the experimentally investigated time scale and supporting the reliability of the corresponding catalytic trends presented in Figure 1. Over the complete kinetic campaign, the catalyst generated approximately 1 × 10^8^ benzene molecules per nominal deposited cluster, further demonstrating sustained catalytic productivity despite repeated changes in temperature and reaction environment.

### 2.3 Morphology Evolution

The structural stability of the clusters was evaluated by *in situ* GIWAXS, and *ex situ* atomic force microscopy (AFM) and scanning electron microscopy (SEM). GIWAXS patterns were acquired at the SIXS beamline at the SOLEIL synchrotron facility (Saint-Aubin, France) in a UHV chamber^40^ exposing the catalysts to ODH feed at up to 1 × 10^–6^ mbar total pressure and for increasing temperature. Despite the difference in pressure with respect to the catalytic experiments, these measurements can provide information on the sintering resistance of the clusters as a function of their composition from room temperature (RT) up to 400 °C. Figure S6a shows the multilayer scattering contribution arising from the thin ZrO_2_ film on the SiO_2_/Si­(100) substrate. The RT WAXS patterns of our catalysts in Figure S6 exhibit no significant difference compared with bare ZrO_2_. Figure 2a shows the scattering patterns acquired at 300 °C under the C_6_H_10_:O_2_ atmosphere (1:1 ratio, *p* = 1 × 10^–6^ mbar), which reveal the emergence of a broad WAXS peak in the range of 28–32 nm^–1^. This range corresponds to the characteristic main wide angle scattering contribution of Cu_*x*_Pd_*y*_ metallic nanoparticles, close to the Cu–Cu and Pd–Pd pair distances in bulk that are between 0.256 and 0.275 nm, respectively. Because subnanometer clusters lack long-range order and therefore do not induce the generation of well-defined diffraction peaks, the appearance of this peak provides an indirect but sensitive signature of thermally induced cluster agglomeration.^40–42^

**Figure 2:**
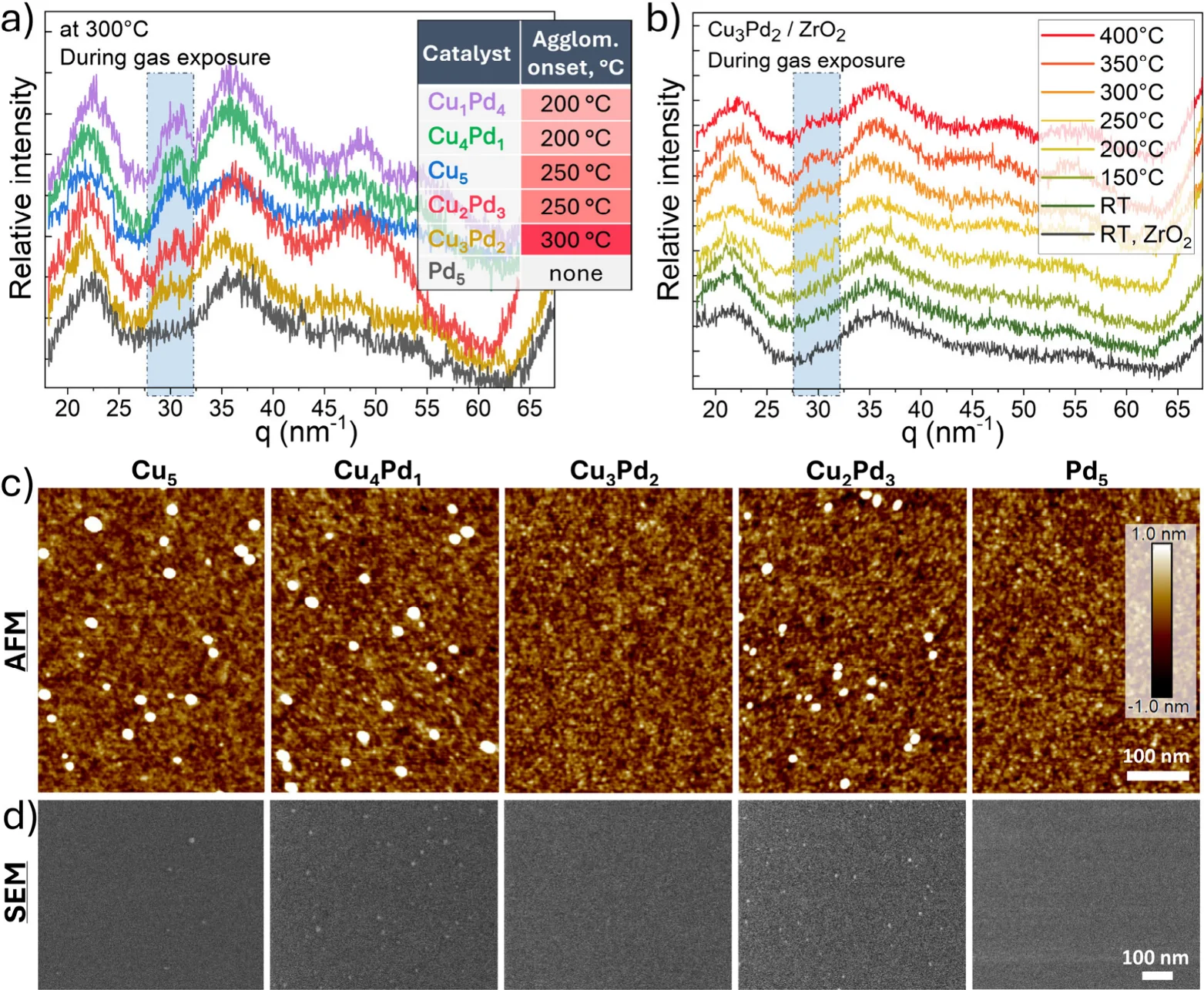
(a) *In situ* GIWAXS patterns acquired at 300 °C under the C_6_H_10_:O_2_ atmosphere (1:1 ratio; total pressure of 1 × 10^–6^ mbar) for all cluster compositions supported on ZrO_2_. (b) Representative diffraction patterns measured for Cu_3_Pd_2_/ZrO_2_ over the entire temperature range from RT up to 400 °C (spectra for all catalysts and all applied conditions are presented in Figure S6). AFM (c) and SEM (d) images acquired for selected samples after catalytic tests performed up to 300 °C.

Clear composition-dependent trends were observed, where Cu_1_Pd_4_ and Cu_4_Pd_1_ exhibited the diffraction reflections, indicating the onset of nanoparticle formation already at 200 °C, consistent with their comparatively low catalytic performance. For Cu_2_Pd_3_, the weak diffraction peak appeared at 250 °C, whereas Cu_3_Pd_2_ remained stable with no detectable reflections up to 300 °C. These behaviors could be partially attributed to the specific atomic arrangement between Cu and Pd close to the equiatomic composition in a condensed matter phase (agglomerated) with 8 Cu nearest neighbors (NN) around each Pd and vice versa as in Cu_2_Pd_3_ and Cu_3_Pd_2_ with respect to 12 NN in a non stoichiometric composition Cu_1_Pd_4_ and Cu_4_Pd_1_. In contrast, Pd_5_ showed no detectable diffraction features at any temperature, indicating exceptional sintering resistance. The representative evolution of the diffraction pattern over the extended temperature range (up to 400 °C) for the most stable bimetallic Cu_3_Pd_2_ is shown in Figure 2b and the corresponding patterns for all catalysts, including the blank ZrO_2_ support, are provided in Figure S6. Moreover, GIWAXS patterns measured under UHV conditions for selected Cu_3_Pd_2_ and Cu_1_Pd_4_ samples (see Figure S6h,i) showed much higher resistance to sintering compared to those exposed to reaction gases. This indicates that cluster agglomeration is not driven solely by thermal effects but also by the chemical environment.

*Ex situ* AFM and SEM measurements (Figure 2c,d) corroborated the GIWAXS trends, confirming partial agglomeration of the clusters after catalytic tests. For Cu_4_Pd_1_ and Cu_5_, nanoislands were observed with lateral diameters of approximately 15 ± 6 nm and 13 ± 8 nm, respectively, and heights of 5 ± 2 nm. Meanwhile, Cu_2_Pd_3_ exhibited slightly smaller nanoparticles, with lateral sizes of 10 ± 4 nm and heights of around 5 ± 1 nm. In contrast, the surfaces of Cu_3_Pd_2_ and Pd_5_ remained smooth, with no detectable nanoparticles, resembling the morphology of the pristine ZrO_2_ support.

The composition-dependent structural evolution observed by GIWAXS is consistent with the catalytic behavior, where the largest changes occurred during the initial temperature ramp, as reflected by the drop in activity within each isothermal step (Figure S1), while the more stable compositions subsequently exhibited comparatively sustained activity. In particular, the comparatively limited structural evolution observed for Cu_3_Pd_2_ is consistent with its sustained catalytic activity during the extended kinetic measurements and subsequent 24 h stability test (Figure S5). For these more stable compositions, the combined results indicate substantial structural and catalytic stability on the experimentally investigated time scale, with any residual structural evolution expected to be limited to changes below the detection sensitivity of the applied characterization methods.

### 2.4 NAP-XPS Characterization

NAP-XPS was employed to investigate surface chemical transformations of the catalyst, with particular focus on the evolution of the chemical states of copper and palladium under ODH reaction conditions. The experiments were conducted in a C_6_H_10_:O_2_ mixture (1:1 ratio) at a total pressure of approximately 1 mbar, with temperatures ranging from RT to 300 °C. These conditions were selected to be comparable to the TPR measurements performed at a total reactant pressure of 6 mbar. In addition, reference measurements were carried out under pure C_6_H_10_ (0.5 mbar) and pure O_2_ (0.8 mbar) at 300 °C in order to disentangle the individual effects of reductive and oxidative environments. The surface chemical composition was monitored at each temperature step by recording the Zr 3d, O 1s, C 1s, and Si 2p core-level spectra.

Figure 3a presents the Cu 2p_3/2_ NAP-XPS spectra of Cu_3_Pd_2_, which is one of the most active and stable bimetallic catalysts. Following exposure to ambient air during sample transfer, the as-received catalyst was initially measured at RT under ultrahigh vacuum (UHV) conditions, and the resulting spectrum was fitted with two components, denoted Cu_A_ and Cu_B_. The Cu_A_ component appears at 933.1 eV, a binding energy (BE) that can be associated with Cu^0^/Cu^+^. These two species cannot be unambiguously distinguished based on the Cu core-level spectra alone and would require a complementary analysis of the Cu LMM Auger region.^43,44^ However, the Auger signals for selected cluster compositions are too weak for reliable analysis because of the low cluster coverage employed to maintain cluster isolation under reaction conditions. In addition, alloying^45,46^ and size effects^47,48^ can introduce further core-level shifts, complicating the interpretation of the experimental spectra as detailed below. Therefore, the interpretation of the spectral components is also discussed in relation to DFT calculations in the following section.

**Figure 3:**
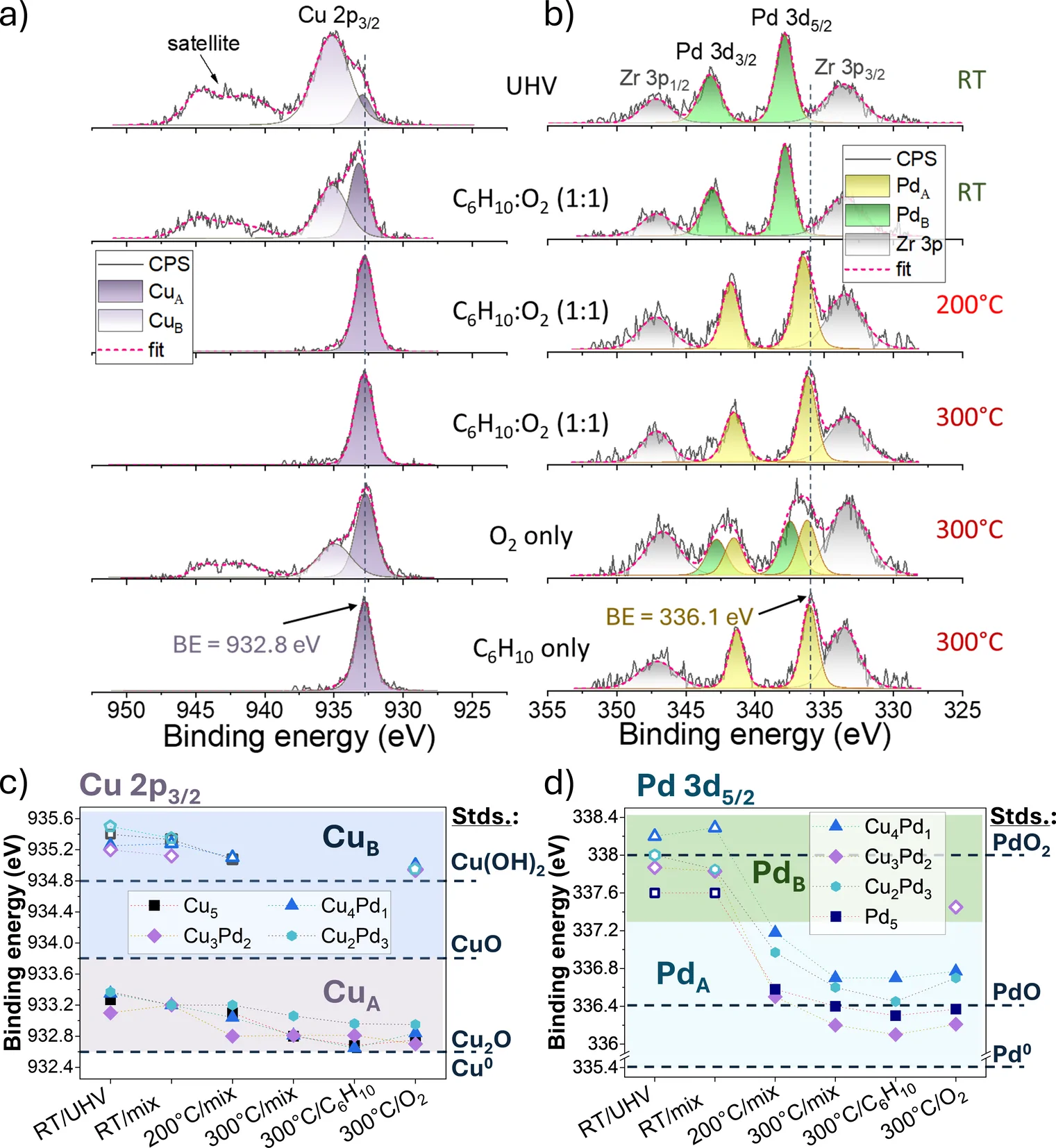
NAP-XPS Cu 2p_3/2_ (a) and Pd 3d (b) spectra acquired from Cu_3_Pd_2_ under different temperature and gas atmosphere conditions. Evolution of BE positions of Cu 2p_3/2_ (c) and Pd 3d_5/2_ (d) for all measured cluster catalysts, where symbol shapes denote cluster composition, while solid and hollow variants of the symbols represent component A and component B, respectively. The binding energies were referenced to the Si 2p_3/2_ core-level of the Si–Si bond with the BE position of 99.2 eV, corresponding to Si 2p peak position of 99.4 eV.^52^ The accuracy of the determined BEs is estimated to be ±0.15 eV, reflecting uncertainties associated with the fitting procedure and the experimental precision of XPS. All NAP-XPS spectra were normalized to their maximum intensity for better comparison.

The Cu_B_ component is located at approximately 935.2 eV. The higher BE with respect to C_A_ indicates that this component is attributed to atoms with higher oxidation states, and the presence of pronounced shakeup satellites at a higher binding energy suggests that Cu_B_ can be attributed to Cu^2+^, most likely originating from Cu­(OH)_*x*_ species.^49–51^ As shown in the topmost spectrum in Figure 3a, the relative intensity of Cu_A_ was relatively low (only about 11%), clearly demonstrating that most of the Cu_3_Pd_2_ clusters oxidize upon air exposure to an oxidation state close to 2+.

Exposure of the sample to the reactant gas mixture at RT resulted in a relative increase of Cu_A_ to about 32%. Upon heating to 300 °C under ODH conditions, both Cu_B_ and the corresponding shakeup satellite completely disappeared, confirming the attribution of Cu_B_ to Cu^2+^. As a result, Cu_A_ remained as the only detectable component at approximately 932.8 eV. This observation indicates a profound chemical transformation in the oxidation state of Cu, which gets partially reduced under ODH conditions. Consequent exposure to 0.9 mbar of pure O_2_ at 300 °C led to the catalyst reoxidation as evidenced by the reemergence of Cu_B_ with the relative abundance of 55% (and 45% of Cu_A_) and of the corresponding satellite structure. Conversely, switching to a pure C_6_H_10_ atmosphere reduced the Cu atoms back, with the Cu_A_ component remaining as the only detectable Cu state. This demonstrates that the copper constituent of the bimetallic cluster exhibits pronounced redox flexibility and responds readily to changes in the chemical environment.

The corresponding Pd 3d spectra of the Cu_3_Pd_2_ cluster, which overlap with the Zr 3p signal from the support, are shown in Figure 3b. The overlap enabled simultaneous monitoring of the chemical state of the support, which remained unchanged under all experimental conditions (see also Zr 3d spectra in Figure S7, where the Zr 3d_5/2_ peak consistently appeared at ∼182.8 eV for all samples). At the same time, the Pd 3d core-level doublet, with a spin–orbit splitting of 5.31 eV and the full width at half-maximum (fwhm) of about 1.5 eV, exhibits redox behavior similar to that observed for Cu. At room temperature under UHV conditions, the Pd 3d_5/2_ peak of the as-received catalyst appears at a BE of 337.9 eV. This value is approximately 1 eV higher than that reported for bulk PdO (around 336.7 eV) and slightly lower than the BE associated with Pd in the +4 oxidation state in PdO_2_, reported at approximately 338 eV.^49^ This component was denoted as Pd_B_ and it is associated with oxidized Pd with a valence between 2+ and 4+. Besides comparison with bulk references, the oxidized state of Pd is confirmed by comparison with UHV XPS studies on *in situ* prepared Pd_*n*_ clusters on TiO_2_, which showed a Pd 3d_5/2_ BE < 336 eV for all clusters with *n* ≤ 10.^53^ Pd_B_ remained stable upon exposure of the sample to the C_6_H_10_:O_2_ gas mixture at RT. However, when the temperature was increased to 200 °C, the Pd 3d doublet shifted toward lower binding energy by approximately 0.9 eV. The shift to lower BE is attributed to the chemical reduction, and the newly formed Pd phase was denoted as Pd_A_. Further increasing the temperature to 300 °C resulted in an additional core-level shift toward lower BE, with the Pd 3d_5/2_ peak appearing at 336.2 eV, which is approximately 0.8 eV higher than that of bulk metallic palladium^49^ and approximately 0.4 eV higher than pristine Pd clusters of comparable size deposited on TiO_2_.^53^ Exposure to O_2_ only resulted in an increase of the fwhm of the Pd spectrum to about 2.6 eV and a shift to higher BE, indicating the coexistence of different oxidation states. Therefore, in this case, the Pd 3d_5/2_ peak was fitted with both Pd_A_ and Pd_B_ components with positions of 336.2 and 337.5 eV, respectively. In contrast, the spectrum acquired during cyclohexene exposure revealed a fwhm of approximately 1.5 eV (consistent with the previous spectral measurements) and was fitted with single Pd_A_ component centered at 336.1 eV.

The NAP-XPS Cu 2p_3/2_ and Pd 3d spectra obtained for all other Cu_*x*_Pd_*y*_ clusters are presented in Figures S8 and S9. These spectra were analyzed by using the same procedure as described for the Cu_3_Pd_2_ sample. As can be seen from Figure 3c,d, which summarizes the evolution of the binding energy positions of the Cu 2p_3/2_ and Pd 3d_5/2_ components for all studied samples, although both metals exhibit an overall trend toward reduction upon heating under ODH conditions, the absolute binding energies and the extent of reduction or oxidation during gas switching depend strongly on the cluster stoichiometry.

In the case of Cu 2p_3/2_, the major differences are observed in Cu_2_Pd_3_, which consistently showed slightly higher binding energies for both Cu_A_ and Cu_B_ components, and Cu_3_Pd_2_, which at 200 °C displayed the lowest Cu_A_ binding energy by about 0.2–0.3 eV with respect to the other clusters. More differences can be observed in the relative abundance of Cu_A_ and Cu_B_ shown in Figure 4 (see Cu 2p_3/2_ spectral series for all catalysts in Figure S8). This analysis highlights how as-received Cu_2_Pd_3_ exhibited the highest fraction of the component associated with the lower oxidation state, Cu_A_ (51%), followed by Cu_4_Pd_1_ (29%), and Cu_5_ and Cu_3_Pd_2_, both at approximately 12%. Exposure to the reactant gas mixture (at RT) led to an increase of Cu_B_ (oxidation state ∼2+) in all samples except Cu_3_Pd_2_, which uniquely showed the inverse trend (an increase of Cu_A_). As discussed for Cu_3_Pd_2_ in Figure 3a, Cu_B_ gradually decreased with increasing temperature for all samples. Cu_B_ and the associated satellite structure typical of Cu^2+^ compounds completely disappear from the Pd-rich clusters (Cu_3_Pd_2_ and Cu_2_Pd_3_) at 200 °C, and at 300 °C for the Cu-rich clusters (Cu_5_ and Cu_4_Pd_1_). At 300 °C under ODH conditions, all clusters show only a Cu_A_ component. The chemical state of the clusters does not further change after the exposure to pure cyclohexene conditions at this temperature. On the contrary, upon subsequent exposure to O_2_, the Cu constituent of all Pd-containing bimetallic clusters underwent reoxidation, as evidenced by the reappearance of the Cu_B_ peak at a BE of 935 eV. Monometallic Cu_5_ appeared unaffected by the low O_2_ pressure of 0.9 mbar. When looking at the Pd 3d core-level spectra (Figure 3b,d), O_2_ exposure led to a change of the Pd chemical state only in Cu_3_Pd_2_, which was fitted with both Pd_A_ and Pd_B_ components, whereas all other clusters retained a single low-BE Pd_A_ state (see comparison of Pd 3d spectra for all catalysts in Figure S9). However, Figure 3d reveals substantial variations in the binding energy of Pd_A_ of up to 0.7 eV across the cluster compositions, suggesting that, while Pd atoms may be present with the same formal oxidation state, their local configuration and electronic structure are not homogeneous among cluster compositions. Under the reactive atmosphere, Cu_3_Pd_2_ consistently displayed the lowest Pd 3d_5/2_ binding energies, whereas Cu_4_Pd_1_ showed the highest values, indicating differences in the local chemical environment of Pd. Such variations may arise from differences in Pd coordination and its interactions with oxygen and neighboring oxidized Cu atoms formed under reaction conditions.

**Figure 4:**
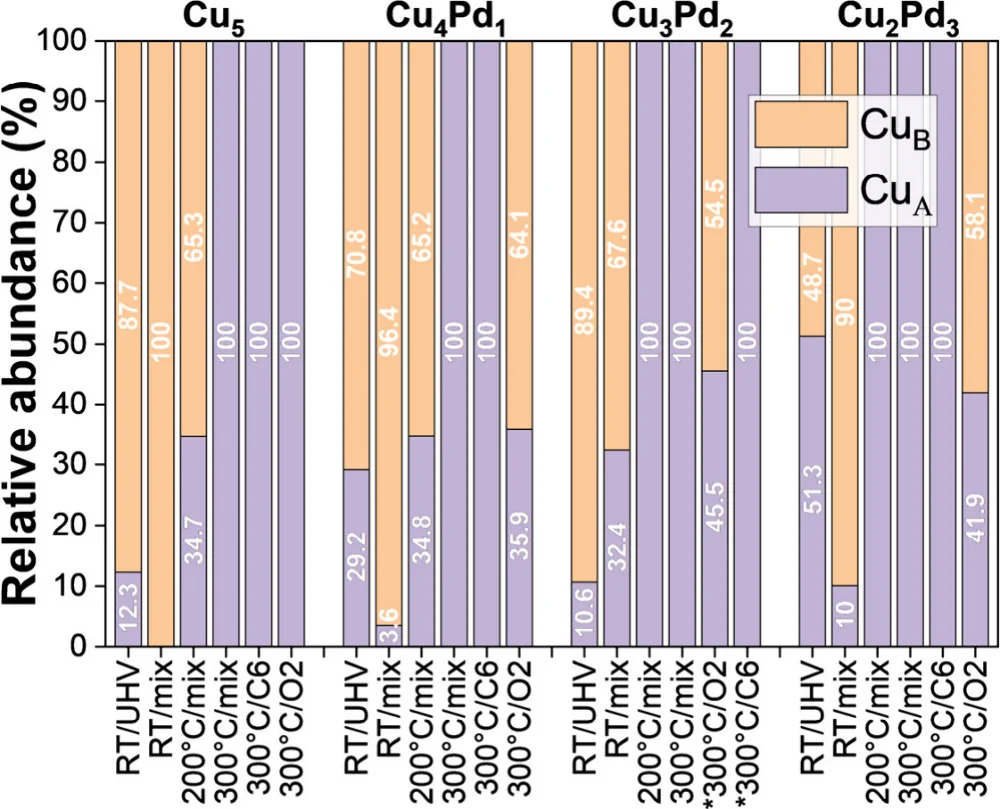
Relative abundance of Cu_A_ and Cu_B_ chemical states derived from the deconvolution of Cu 2p_3/2_ spectra under different NAP-XPS conditions. A standard uncertainty of ±10% was assumed for the evaluated Cu_A_/Cu_B_ ratios, reflecting the typical experimental/fitting precision of the XPS. All data are presented in the order of measurement, while conditions marked with an asterisk in the graph indicate points where the sequence deviates from this order.

While it is generally accepted that for Cu and Pd a positive core-level shift, i.e., farther from the Fermi level, is associated with an increase in the oxidation state,^44,49^ in the case of size-selected clusters these fundamental correlations may break down^54^ due to a complex combination in initial-/final-state effects,^53–55^ high degree of fluxionality,^33,56^ and strong cluster-support interactions.^57,58^ Anderson et al. reported that Pd clusters deposited *in situ* exhibit core-level shifts of up to 0.6 eV toward higher binding energy relative to the bulk metal,^53^ while Watanabe and co-workers reported comparable trends for Pt clusters, showing that, in addition to the initial- and final-state contributions, BE shifts further correlate with the degree of surface confinement and the resulting cluster geometry.^59,60^ As demonstrated in our previous work on platinum clusters, large BE shifts, comparable to those observed here (up to +0.8 eV), can be attributed to partially oxidized states featuring intermediate δ+ species arising from charge transfer at the metal–support interface, thereby modifying the cluster electronic structure, apparent oxidation state, and catalytic activity.^57^ To address the complexity of these size-related effects and achieve an atomistic understanding of the experimental results, we relied on machine-learning interatomic potentials (MLIP) and DFT calculations.

### 2.5 DFT and MLIP-Calculated Structural and Electronic Properties

The first step of the calculations focused on the configurational distributions of bimetallic Cu_*x*_Pd_*y*_ clusters supported on model amorphous ZrO_2_ under experimentally relevant environments. The intrinsic electronic and structural flexibility of subnanometer clusters enable them to adopt multiple distinct configurations on the support, leading to pronounced structural fluxionality.^32,61^ Previous theoretical studies have established that the ensemble of thermally accessible isomers, as well as its evolution with adsorbate coverage and temperature, governs binding-site availability, adsorption modes, and reactivity.^30,62^ Because the NAP-XPS and catalytic TPR measurements were performed under different pressure regimes, we used two related but distinct computational environments. MLIP-based global optimization under the NAP-XPS conditions was used to benchmark the calculated electronic structures against the measured core-level shifts. After this spectroscopic benchmark, cluster-specific reaction environments derived from the TPR measurements at 300 °C were used to sample the ensembles shown in Figure 5 and subsequently used for cyclohexene adsorption analysis. The corresponding partial pressures of C_6_H_10_, C_6_H_6_, O_2_, H_2_O, and H_2_ used for each cluster composition are reported in Table S1.

**Figure 5:**
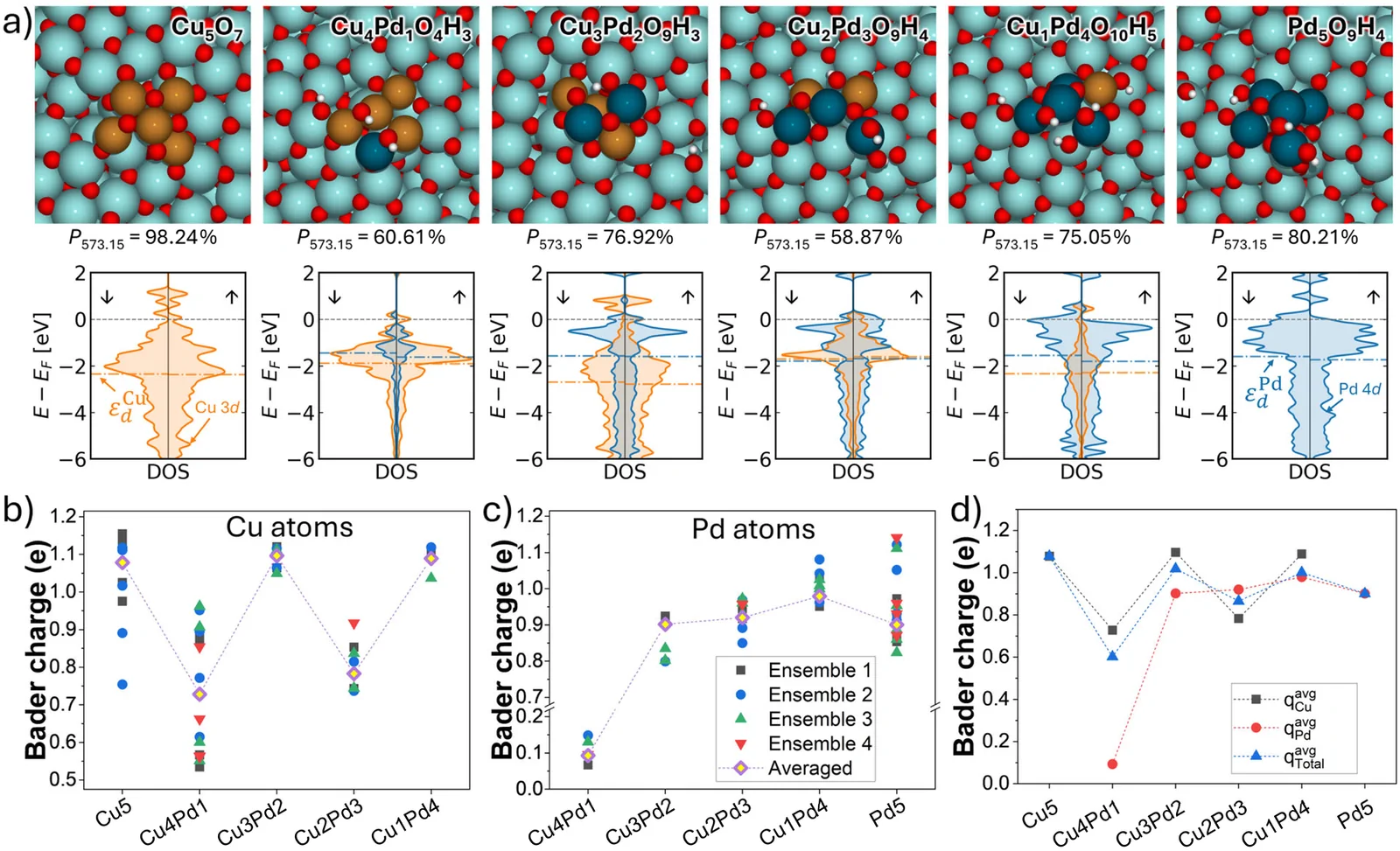
(a) Most Boltzmann-populated Cu_*x*_Pd_*y*_ pentamer structures on model amorphous ZrO_2_ under cluster-specific TPR-derived ODH environments at 300 °C. The Boltzmann population at the experimental temperature is indicated below each structure. The corresponding element-projected density of states (pDOS) for Cu 3d (yellow) and Pd 4d (blue) states are shown below, with the d-band centers indicated. Ensemble-resolved Bader charge distributions from the TPR-derived ensembles for (b) Cu and (c) Pd are shown together with their ensemble-averaged values. (d) Ensemble-averaged Bader charges for Cu and Pd, together with the total Bader charge values of the respective pentamer clusters. See Table S1 for the gas-phase partial pressures employed in the simulations.

Figure 5a summarizes the most Boltzmann-populated structures for all cluster compositions supported on amorphous ZrO_2_, together with their corresponding projected densities of states (pDOS). Cu_5_ exhibits the lowest ensemble diversity, with only two thermally accessible isomers and a Boltzmann population dominated by a small number of configurations. In contrast, the bimetallic CuPd pentamers and Pd_5_ show higher structural fluxionality, with three to four distinct isomers contributing significantly to the thermally accessible ensembles (see Figure S10 for all ensemble configurations). This increased multiplicity indicates that Pd-containing clusters can sample a broader range of geometries under the reaction conditions. Because binding-site availability, adsorption modes, and reactivity in supported subnanometer clusters are governed by the thermally accessible ensemble and its evolution with adsorbate coverage and temperature,^30,31,63^ the broader configurational space of Pd-containing clusters is expected to provide a more diverse set of local metal–oxygen motifs. This diversity is relevant for the subsequent cyclohexene adsorption analysis, where the arrangement of oxygen around the cluster becomes important. Conversely, the limited ensemble diversity of Cu_5_ together with its distinct adsorption environment discussed below, is consistent with its poor catalytic performance observed in Figure 1.

The most Boltzmann-populated structures also exhibit composition-dependent O and H adsorbate coverages. The Pd-rich Cu_*x*_Pd_*y*_ clusters (Pd ≥ 2) display comparable adsorbate loadings, typically with 9–10 O atoms and 4–5 H atoms bound to the most populated pentamer structures. Together with the Pd-derived states remaining near the Fermi level in the pDOS, this indicates that Pd-containing clusters can stabilize highly oxygenated configurations while retaining appreciable Pd-derived electronic states. Thus, under reaction conditions, Pd in these clusters is best described as oxygen-coordinated and partially positively charged, yet electronically active. In contrast, Cu_4_Pd_1_ accommodates substantially fewer adsorbates, with a representative stoichiometry of Cu_4_Pd_1_O_4_H_3_. This lower adsorbate loading likely contributes to the smaller positive charge accumulated on the metal atoms in Cu_4_Pd_1_, indicating that the ensemble-averaged charge state is governed not only by Cu/Pd composition but also by the adsorbate stoichiometry stabilized under the corresponding reaction environment. The monometallic Cu_5_ pentamer represents another distinct case: although it stabilizes a relatively high oxygen coverage, with 7 O atoms in the most populated structure, it does not bind H under these conditions. The electronic description of these oxygenated clusters was further benchmarked through Cu 2p_3/2_ core-level-shift calculations under NAP-XPS-matched conditions (Figure S11). The analysis, discussed in Section S1 of the Supporting Information, shows good agreement between theory and experiment.

To clarify how these composition-dependent ensembles differ electronically, we next performed an ensemble- and element-resolved DFT analysis of the Cu_*x*_Pd_*y*_ pentamers under TPR-derived ODH environments. The pDOS analysis in Figure 5a shows that the Pd- and Cu-derived d states respond differently to changes in cluster composition. The Pd-derived 4d band centers ε_d_^Pd^ are relatively less sensitive to composition than the Cu-derived 3d band center, although the detailed Pd pDOS line shapes vary across the series. In contrast, the Cu-derived 3d states exhibit more pronounced composition-dependent shifts and changes in spectral shape. These trends point to a cooperative oxygenated electronic state in which Pd retains substantial DOS near *E*_F_ despite oxygen coordination and partial positive charging, while Cu provides a more composition-sensitive electronic response associated with changes in oxygen binding and charge redistribution. This combined Pd–Cu response provides an electronic basis for the reaction-relevant adsorption motifs discussed below.

The Bader charge analysis in Figure 5b–d further quantifies how charge redistribution varies across the TPR-derived ensembles shown in Figure 5a. For most Pd-containing compositions, both Cu and Pd atoms carry positive charges in a broadly comparable range, indicating that charge transfer is not localized exclusively on one metal component. The main exception is Cu_4_Pd_1_, for which both the Cu and Pd charge distributions are shifted to substantially smaller positive values than those of the other bimetallic and Pd-rich clusters. This behavior is consistent with the lower O/H adsorbate loading of the dominant Cu_4_Pd_1_ structure rather than indicating an intrinsic suppression of charge transfer by composition alone. More generally, the ensemble-averaged Bader charges should be interpreted as reflecting the combined effects of cluster composition, adsorbate stoichiometry, local metal–oxygen coordination, and the cluster-specific gas environment used in the TPR-derived simulations. Together with the pDOS trends, this Bader analysis supports the picture of cooperative oxygenated Cu–Pd ensembles, in which both metals participate in charge redistribution, while the magnitude of the apparent charge transfer is strongly coupled to the O/H adsorbate environment stabilized under reaction conditions.

The Cu charge distributions in Figure 5b show stronger composition- and ensemble-dependent variability than the corresponding Pd distributions in Figure 5c. The Cu Bader charges span a broader range across the TPR-derived ensembles, indicating substantial heterogeneity in the local Cu environments. This variability follows the changes in Cu-derived pDOS features and Cu 3d band centers in Figure 5a but should not be attributed to composition alone. Because the clusters differ in O/H adsorbate loading and in the local placement of oxygenated species, the Cu charge response is most directly associated with local Cu–O/Cu–OH coordination, adsorbate stoichiometry, and cluster geometry. Small-cluster electron-counting effects may also contribute to the composition-dependent electronic response, as reported for ultrasmall Cu-containing clusters,^27,64–68^ but under ODH conditions, this intrinsic electronic sensitivity is coupled to adsorbate-induced charge redistribution. Thus, Cu participates in tuning the local metal–oxygen environment and charge distribution of the oxygenated CuPd clusters, while the resulting electronic structure reflects the combined effects of composition, adsorbate coverage, and local coordination. We next examine how this ensemble-dependent electronic structure is reflected in cyclohexene adsorption behavior.

### 2.6 Cyclohexene Adsorption

Building on the spectroscopically benchmarked electronic description and the TPR-derived oxygenated ensembles, we examined how the local oxygen environment influences the cyclohexene adsorption. We focused on representative compositions, Cu_5_, Cu_2_Pd_3_, and Pd_5_, which span the inactive Cu-only limit, the highly active bimetallic regime, and the highly active Pd-only limit. For each composition, cyclohexene adsorption was evaluated on the thermally accessible oxygenated structures sampled under the corresponding TPR-derived ODH environment (Table S1), and the representative adsorption motifs are shown in Figure 6.

**Figure 6:**
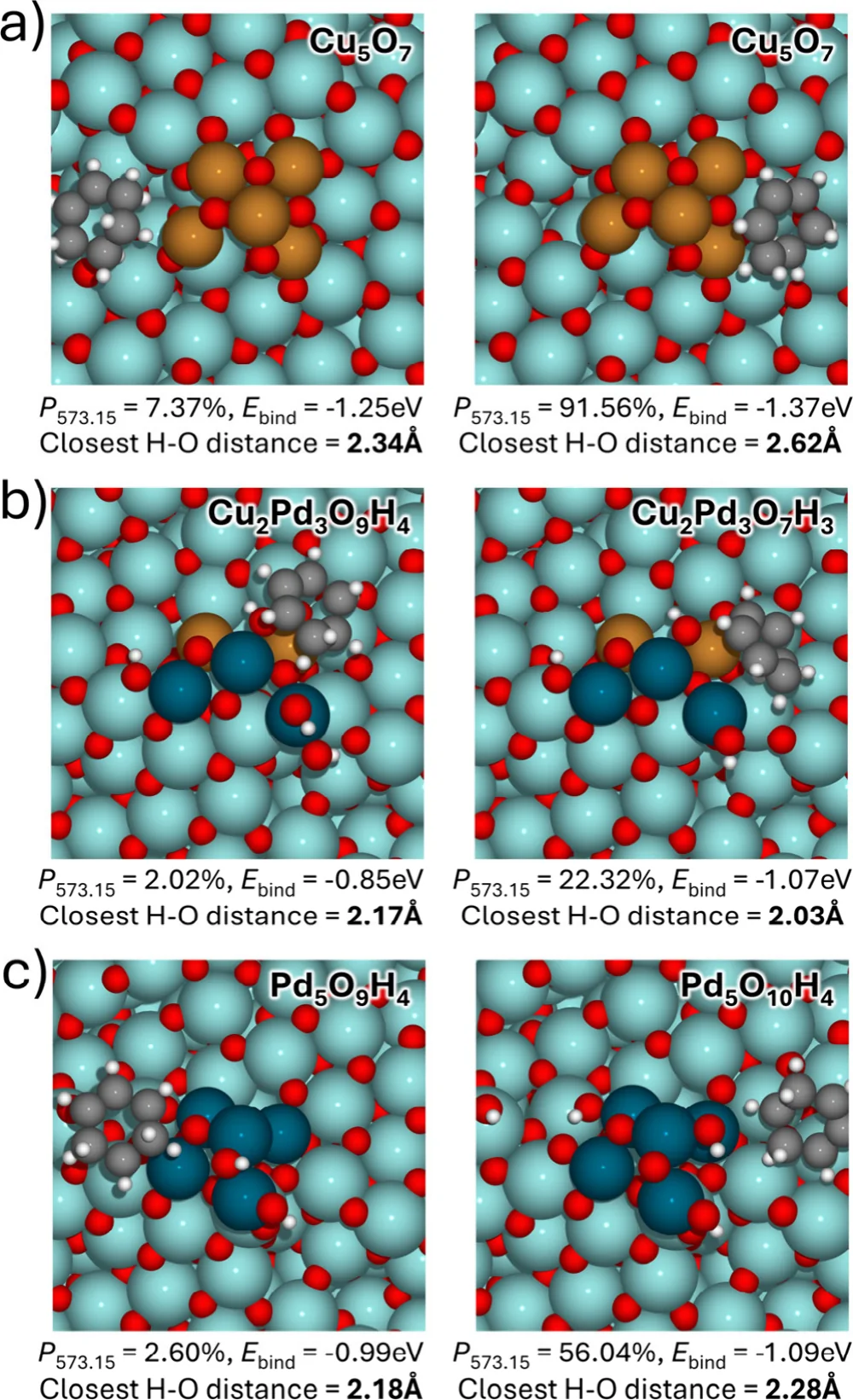
Representative cyclohexene (C_6_H_10_) adsorption geometries selected from the thermally accessible oxygenated ensembles of (a) Cu_5_, (b) Cu_2_Pd_3_, and (c) Pd_5_ supported on ZrO_2_ under TPR-derived ODH environments. The selected motifs correspond to configurations with short H­(cyclohexene)–O contacts among the sampled adsorption structures. The H–O distances shown indicate the closest distance between a cyclohexene H atom and a nearby O/OH species on the oxygenated cluster. Adsorption energies are defined as *E*_bind_ = *E*_cluster/ZrO_2_+C_6_H_10__ – *E*_cluster/ZrO_2__ – *E*_C_6_H_10__, where more negative values indicate stronger cyclohexene binding. The TPR feed corresponds to a C_6_H_10_:O_2_ ratio of 1:1 at a total pressure of ∼1.1 bar, with ∼6 mbar total reactant partial pressure diluted in He; the cluster-specific gas compositions used in the calculations are provided in Table S1.

Figure 6 shows that cyclohexene adsorption is governed not only by adsorption strength, but also by the identity and local coordination of nearby oxygenated species. Cu_5_ binds cyclohexene relatively strongly, with adsorption energies of −1.25 and −1.37 eV. However, the nearest oxygen species in these structures are either a 3-fold Zr_2_Cu-bound interfacial O atom or a 3-fold Cu-bound O atom, with H–O distances of 2.34 and 2.62 Å, respectively. In contrast, the active Cu_2_Pd_3_ and Pd_5_ clusters place Pd-associated oxygenated species near cyclohexene H atoms. For Cu_2_Pd_3_, the nearest oxygen species is either a onefold Pd-bound OH group or a Cu–Pd bridge-bound OH group, with closest H–O distances of 2.17 and 2.03 Å, respectively. For Pd_5_, the nearest oxygenated species is a 3-fold Pd-bound O atom or a onefold Pd-bound OH group, with H–O distances of 2.18 and 2.28 Å, respectively.

These adsorption geometries indicate that the presence of oxygen alone is not sufficient to rationalize activity. Rather, the local coordination of O/OH species appears important for forming C–H···O contacts relevant to C–H activation. The active Pd-containing clusters generate Pd-bound or Cu–Pd-bridged O/OH motifs that place oxygenated species in proximity to cyclohexene H atoms, whereas Cu_5_ primarily involves Cu-bound or support-associated oxygen motifs. The H–O distance is therefore used here as a geometric descriptor of hydrogen-transfer proximity, not as evidence for a fully developed hydrogen-abstraction transition state. This distinction is important because Cu_5_ can bind cyclohexene strongly yet does not provide the same Pd-associated oxygen environment observed for the active clusters.

### 2.7 DFT/MLIP Calculations of C–H Activation

To test whether the short C–H···O contacts identified in Figure 6 provide kinetically accessible activation motifs, we calculated the first C–H activation for two populated oxidized configurations of each of Cu_2_Pd_3_, Pd_5_, and Cu_3_Pd_2_; Cu_3_Pd_2_ directly matches the catalyst used for the kinetic measurements. In all six pathways, H transfers from C_6_H_10_ to cluster-bound O through a bridging C–H···O transition structure. The pathways were obtained with a shared reaction-path-refined MLIP using adaptively densified NEB bands and climbing-image refinement of the H-transfer region, and each transition state was confirmed by a single imaginary mode in the adsorbate partial Hessian (170–1015 cm^–1^).

At 573.15 K, the adsorbed-state ΔG^‡^ values are 0.12 and 0.34 eV for Cu_2_Pd_3_, 0.34 and 0.45 eV for Pd_5_, and 0.36 and 1.09 eV for Cu_3_Pd_2_. Cyclohexene adsorption is weak and reversible: Δ*G*_ads_ is positive for five configurations (+0.30 to +0.46 eV) and is −0.16 eV for Cu_2_Pd_3_(O_7_H_3_); positive Δ*G*_ads_ indicates low but finite coverage. The corresponding gas-referenced effective barrier is Δ*G*_ads_ + Δ*G* ^‡^.

For the representative pathways in Figure 7, the effective barriers are 0.45, 0.73, and 0.82 eV for Cu_2_Pd_3_, Pd_5_, and Cu_3_Pd_2_, respectively. These pathways illustrate the composition dependence within thermally populated oxidized configurations; because adsorption-mode populations were not evaluated, they are not assigned as kinetically dominant. The accessible Cu_3_Pd_2_ pathway is consistent with the measured low-temperature activity of the same composition, while the full set demonstrates a strong composition- and O-site dependence. Because these calculations address only the first H-transfer step, they identify a plausible rate-relevant O-assisted pathway but do not establish the global rate-determining step, experimental activity ranking, or H_2_/H_2_O branching. The MLIP-level energies have an approximate 0.1–0.2 eV uncertainty for this soft, weakly adsorbed system; accordingly, mechanistic accessibility and configuration dependence are emphasized over fine numerical differences. Full computational details, results, and the combined six-panel NEB profile are provided in Section S2, Table S2, and Figure S12.

**Figure 7:**
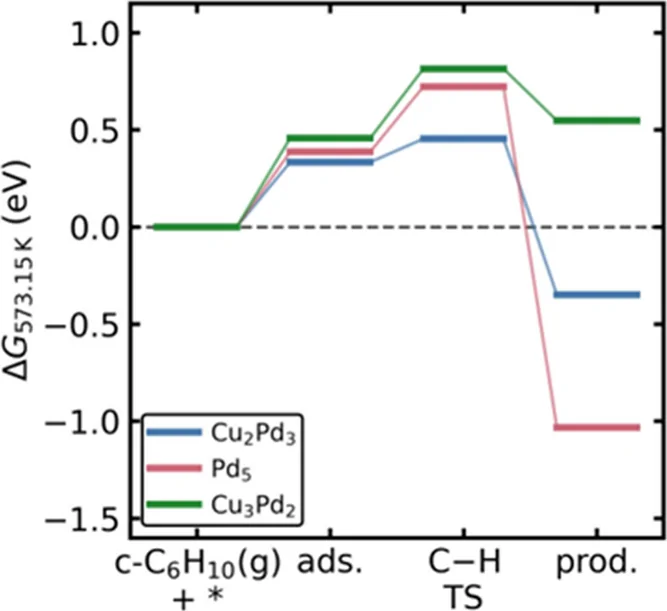
Free-energy landscape at 573.15 K for the first O-assisted C–H activation along representative pathways sampled from thermally populated oxidized configurations of Cu_2_Pd_3_, Pd_5_, and Cu_3_Pd_2_. Energies are referenced to gas-phase c-C_6_H_10_ plus the bare supported cluster; the gas-referenced effective barrier is Δ*G*_ads_ + Δ*G* ^‡^.

### 2.8 Summary and Theory–Experiment Comparison

The superior catalytic performance of Pd-containing clusters supported on ZrO_2_ under nominal ODH conditions (C_6_H_10_:O_2_, 1:1) can be rationalized by a combination of electronic, structural, and kinetic factors unique to this catalyst system. Ensemble analysis shows that Pd-containing clusters accommodate substantial oxygen coverage while retaining appreciable Pd-derived density of states near the Fermi level, indicating an oxygen-coordinated and partially positively charged, yet electronically active, Pd environment. At the same time, Cu contributes a composition-sensitive electronic response associated with oxygen binding and charge redistribution within the oxygenated Cu–Pd ensembles. These theoretical observations further support the NAP-XPS results, confirming that under ODH active conditions the clusters exist in partially oxidized states, as reflected by the binding-energy positions of the Cu_A_ and Pd_A_ components (Figure 3c,d), although additional positive shifts arising from cluster-size effects cannot be excluded. Consistently, Cu exhibits pronounced redox flexibility upon changes in the gas environment, as evidenced by its strong response to pure oxygen or cyclohexene and by the emergence or disappearance of the Cu_B_ component, associated with a higher oxidation state close to 2+, while Pd shows a considerably weaker redox response, with the higher oxidation-state Pd_B_ species observed only for Cu_3_Pd_2_ under O_2_ exposure at 300 °C. According to DFT, Pd-containing clusters also exhibit higher ensemble diversity than Cu_5_, accessing multiple thermally populated geometries under reaction conditions. DFT adsorption analysis further shows that the active Cu_2_Pd_3_ and Pd_5_ clusters form Pd-bound or Cu–Pd-bridged O/OH motifs that place oxygenated species near cyclohexene H atoms, providing geometries relevant to C–H activation.^69^ This structural and electronic diversity helps rationalize efficient dehydrogenation by combining electronically active Pd-containing motifs with oxygenated local environments suitable for cyclohexene activation. Activity normalized per Pd atom exhibits substantially smaller variation across the cluster compositions, with Cu_2_Pd_3_, Cu_3_Pd_2_, and Pd_5_ showing nearly comparable values, highlighting the central role of Pd-containing motifs rather than a purely composition-independent Pd site. In contrast, Cu_5_ lacks Pd-associated oxygenated motifs and predominantly forms Cu-bound or support-associated oxygen environments, leading to adsorption geometries less favorable for hydrogen-transfer proximity despite relatively strong cyclohexene binding. Separately, the GIWAXS results (Figures 2a and S6) show that the most active compositions also exhibit delayed or absent emergence of metal–metal diffraction features, indicating resistance to agglomeration. In contrast, Cu_4_Pd_1_ and Cu_1_Pd_4_ exhibit an earlier appearance of metallic diffraction signatures, indicative of reduced sintering resistance, as also evidenced by the significant drop in catalytic activity with each consecutive temperature ramp (see Figure S1). This behavior is qualitatively consistent with literature reports indicating that certain Cu–Pd compositions can be thermodynamically metastable, where compositional asymmetry may promote structural heterogeneity and facilitate agglomeration.^70^

Overall, the electronic trends in Figure 5 provide a composition-dependent picture that is qualitatively consistent with the catalytic behavior observed experimentally. Cu_4_Pd_1_ is distinct in both its lower O/H loading and smaller positive metal charges, whereas the Pd-rich clusters retain appreciable Pd-derived states near *E*_F_, together with more oxygenated local environments. These differences suggest that catalytic performance is governed not by composition alone but by the coupled effects of oxygen coverage, charge redistribution, and the ability of Pd-containing motifs to stabilize reaction-relevant O/OH environments.

Within this context, the experimental observation that the reaction is strongly suppressed in the complete absence of O_2_ indicates that oxygen is required to initiate low-temperature dehydrogenation and establish the catalytically relevant state of the clusters. A minimum O_2_ concentration is necessary to trigger cyclohexene dehydrogenation at low temperatures, as shown in Figure 1 for different gas feeds. At the same time, oxygen-lean conditions promote molecular H_2_ release and suppress H_2_O formation, resulting in a H_2_ selectivity of up to 99%. Higher O_2_ availability increases the extent of oxidative hydrogen removal toward H_2_O formation, although H_2_ release remains predominant even under stoichiometric ODH conditions (see Figure 1). This trend is consistent with previous reports that oxygen activation becomes more effective at elevated temperatures.^25,37^ Although DFT calculations were not explicitly performed under oxygen-lean conditions, the TPR-derived ODH ensembles indicate that adsorbed O/OH species can stabilize cyclohexene adsorption motifs in which oxygenated species are positioned near cyclohexene H atoms. These motifs provide geometries relevant to C–H activation without requiring excessive cyclohexene overbinding, consistent with the experimentally observed oxygen-promoted yet H_2_-selective dehydrogenation behavior.

The kinetic measurements described in section 2.2 provide an independent experimental constraint on this ensemble-based picture. The positive fractional dependence of benzene formation on both C_6_H_10_ and O_2_ demonstrates that the rate is sensitive to the availability of both reactants, while the temperature dependence of the apparent reaction orders indicates that this sensitivity evolves with reaction conditions. The opposite feed-composition dependences of the apparent activation energy further show that the observed kinetics cannot be represented by a single invariant elementary barrier. Instead, the results are consistent with a reaction-condition-dependent catalytic ensemble in which hydrocarbon coverage, O/H loading, and cluster oxidation state influence the effective kinetic landscape. The new first-step free-energy calculations show that O-assisted H transfer is accessible on populated Cu_2_Pd_3_, Pd_5_, and Cu_3_Pd_2_ configurations; the representative gas-referenced effective barriers, Δ*G*_ads_ + Δ*G* ^‡^, are 0.45, 0.73, and 0.82 eV, respectively. The accessible Cu_3_Pd_2_ pathway provides a direct theoretical link to the composition used for the kinetic measurements. However, the measured *E*_app_ is a macroscopic quantity and is not assigned to a single calculated transition state; the six final NEB profiles compiled in Figure S12 are reported as MLIP-level results and are used to establish trends and composition dependence rather than an experimental activity ranking.

The kinetic hydrogen-product branching further strengthens this interpretation. Increasing C_6_H_10_ availability favors H_2_ formation, whereas increasing O_2_ availability and temperature shift the removed-hydrogen distribution toward H_2_O. These observations provide direct experimental evidence that H–H recombination competes with oxidative removal of surface hydrogen. The first-step calculations establish an O-assisted route for H transfer to cluster-bound O, while the reaction-conditioned Pd-containing ensembles provide configurations compatible with both adsorbed H and O/OH species. Because H migration, H–H recombination, and competing H_2_/H_2_O-forming pathways were not calculated, the computational results do not determine the observed hydrogen-product branching.

The observed product distribution cannot be explained solely by a classical Mars–van Krevelen (MvK)-type water-forming pathway,^71,72^ in which surface hydroxyls recombine to form water. Instead, the present system appears to operate in a regime where H–H recombination can compete with oxidative hydrogen removal. This behavior may be promoted by stepwise dehydrogenation of cyclohexene,^9,17,28,73^ which can generate multiple H atoms in close temporal and spatial proximity. On fluxional Pd-containing clusters, such hydrogen species may access configurations favorable for H–H recombination, especially under oxygen-lean conditions and at low reaction temperatures, whereas increasing the oxygen chemical potential increases the availability of O/OH-containing environments capable of converting surface hydrogen toward H_2_O.

The present experimental and theoretical results therefore support a reaction-condition-dependent competition between molecular H_2_ release and oxygen-assisted hydrogen removal while defining the limits of the mechanistic assignment. The six calculated pathways identify the first O-assisted C–H activation through a bridging C–H···O saddle and show strong composition- and O-site dependence, but they do not establish this step as the global rate-determining step. Resolving the complete catalytic sequence and the H_2_/H_2_O branching would require combinatorial sampling of later C–H cleavages, H migration and H–H recombination, and competing H_2_/H_2_O pathways across multiple fluxional O/H ensembles, followed by microkinetic analysis. Such a complete network constitutes a separate mechanistic study beyond the scope of the present work.

Taken together, the combined experimental and theoretical results provide a coherent picture linking the electronic structure, oxygenated adsorption motifs, oxidation-state evolution, reaction kinetics, and catalytic performance. Pd-containing clusters accommodate oxygen while retaining electronically active Pd-derived states near the Fermi level, and the populated Cu_2_Pd_3_, Pd_5_, and Cu_3_Pd_2_ configurations provide accessible first O-assisted H-transfer pathways. The positive O_2_ reaction order and increased *E*_app_ under oxygen-lean conditions support the role of oxygen in establishing the kinetically relevant cluster environment, while the measured H_2_/H_2_O branching demonstrates that oxygen availability also controls the partitioning of the removed hydrogen. The calculations support the accessibility and composition dependence of the first O-assisted C–H activation, whereas the experimental kinetics and product analysis provide direct evidence for rate dependence and hydrogen-product branching. More broadly, the identified role of O_2_ availability in controlling H_2_/H_2_O branching during dehydrogenation may be relevant to a wider range of hydrogen-release reactions, including those involving saturated alkanes, with direct implications for hydrogen carrier technologies.

## 3 Conclusions

We demonstrate that subnanometer Cu_*x*_Pd_*y*_ pentamer clusters supported on ZrO_2_ form a highly active and selective class of dehydrogenation catalysts, that operate at low temperatures. Pd_5_ clusters exhibited the highest catalytic activity toward benzene formation, with activity already observed at 150 °C, followed by bimetallic Cu_2_Pd_3_ and Cu_3_Pd_2_ clusters, whereas pure Cu_5_ clusters were inactive. Remarkably, H_2_ was the dominant hydrogen-containing product even under stoichiometric ODH conditions, exceeding H_2_O formation, while an oxygen-lean feed further favored H_2_ production, reaching rates of up to 213 molec.·atom^–1^·s^–1^ and H_2_ selectivity above 99% under selected conditions. In contrast, removal of O_2_ strongly suppressed the reaction, demonstrating that oxygen is required not only to establish the catalytically relevant cluster state but also to control H_2_/H_2_O branching. Kinetic analysis of Cu_3_Pd_2_ revealed positive fractional apparent orders in both C_6_H_10_ and O_2_ and an effective apparent activation energy of approximately 66 kJ·mol^–1^ under the standard feed. The dependence of the apparent kinetics and H_2_/H_2_O branching on temperature and feed composition further demonstrates that reactant coverage and oxygen availability directly influence the reaction-relevant catalyst state.

The spectroscopic, structural, and theoretical results provide a consistent picture of the factors governing this behavior. NAP-XPS reveals an overall trend toward reduction upon heating under ODH conditions for both metals, with the resulting electronic state strongly dependent on cluster stoichiometry. Cu within the clusters exhibits pronounced redox flexibility in response to oxygen availability, whereas Pd accommodates oxygen in a partially oxidized form while retaining electronically active Pd-derived states near the Fermi level, as supported by DFT calculations. DFT further shows that the most active Cu_2_Pd_3_ and Pd_5_ ensembles stabilize Pd-bound or Cu–Pd-bridged O/OH motifs that position oxygenated species near cyclohexene H atoms, providing adsorption geometries favorable for C–H activation. In contrast, Cu_5_ binds cyclohexene relatively strongly but lacks comparable Pd-associated oxygenated motifs, consistent with its poor catalytic performance. DFT/MLIP free-energy calculations further identify accessible first O-assisted C–H activation pathways for populated Cu_2_Pd_3_, Pd_5_, and Cu_3_Pd_2_ configurations through bridging C–H···O transition structures. These results support a mechanistic role of cluster-bound oxygen in the initial H-transfer step without assigning this step as globally rate determining or as the origin of H_2_/H_2_O branching. In parallel, *in situ* GIWAXS shows that the most active compositions are comparatively resistant to structural transformation, whereas Cu_4_Pd_1_ and Cu_1_Pd_4_ undergo earlier agglomeration and deactivation. Comparison of reactive-gas and UHV measurements further shows that this structural evolution is governed by the chemical environment as well as temperature.

Overall, the catalytic behavior of size-selected Cu_*x*_Pd_*y*_ clusters is governed by three interdependent factors: (i) ensemble multiplicity arising from fluxionality, which provides diverse accessible adsorption geometries; (ii) composition-dependent electronic response, where partially oxidized Pd-containing clusters retain electronically active Pd-derived states and Cu contributes to oxygen binding and charge redistribution; and (iii) the formation of local O/OH motifs favorable for O-assisted C–H activation. This Cu–Pd synergy combines Pd-centered reactivity with the redox tunability of Cu, enabling highly active bimetallic compositions while providing control over the reaction-relevant cluster state and more efficient utilization of Pd. Most importantly, the observation that H_2_ can remain the dominant hydrogen-containing product under oxidative conditions challenges the conventional description of ODH as an exclusively water-forming process and identifies oxygen availability as a means of directing H_2_/H_2_O branching toward selective hydrogen release under mild conditions. Together with our previous demonstration of selective cyclohexane-to-benzene dehydrogenation on atomically precise supported clusters and realistic powder-based catalysts,^19,34,36^ these findings motivate the extension of this concept to more demanding saturated hydrocarbons and technologically relevant LOHC systems, including the methylcyclohexane/toluene pair.

## 4 Experimental Methods

### 4.1 Catalysts Preparation

#### 4.1.1 Atomic Layer Deposition (ALD)

ALD was utilized to prepare thin ZrO_2_ films. As substrates, n-type P-doped Si wafers from Siegert (⟨100⟩, resistivity 1–5 ohm cm, thickness 525 ± 10 μm, one-side polished) were used. The wafers were cut into 20 × 22 mm chips for catalytic tests and 8 × 6.5 mm/10 × 10 mm chips for *ex situ* and *in situ* characterization. Prior to ALD deposition, Si chips with a native oxide film of about 2 nm thickness were ultrasonically cleaned three times for 5 min in ethanol (Sigma-Aldrich, ≥99.5%, ACS reagent, 200 proof) using a Branson 2800 ultrasonic cleaner. The thickness of the native SiO_2_ layer was measured using a J.A.Woollam α-SE variable angle spectroscopic ellipsometer (Lincoln, NE) at an incident angle of 70° over the 380–900 nm wavelength range.

ZrO_2_ films were prepared in a viscous-flow benchtop ALD reactor (Gemstar-6, Arradiance) at a pressure of ∼1.3 mbar. Tetrakis­(dimethylamino)­zirconium­(IV) (TDMAZ, Zr­[N­(CH_3_)_2_]_4_, Sigma-Aldrich, electronic grade, ≥99.99% trace metals basis) and Deionized H_2_O were used as precursors, and ultra high purity N_2_ (Nexair, 99.999%) served as the carrier gas. The N_2_ was further purified prior to entering the reactor using a Supelco gas purifier (Sigma-Aldrich). ZrO_2_ films were deposited using 7 ALD cycles consisting of alternating exposures of TDMAZ and H_2_O. Each cycle followed a pulse-purge sequence: TDMAZ pulse (50 ms), N_2_ purge (12 s), H_2_O pulse (100 ms), N_2_ purge (12 s). The reaction chamber temperature was maintained at 140 °C.

An additional batch of ZrO_2_ films for the GIWAXS experiment was prepared in a Fiji 200 ALD reactor (Ultratech/Cambridge NanoTech) at a pressure of ∼0.3 mbar. TDMAZ precursor (99.99%, Strem Chemicals) was used as the zirconium precursor. High-purity deionized water (H_2_O) was used as the co-reactant. TDMAZ cylinder was maintained at 75 °C to obtain a sufficiently high vapor pressure. ZrO_2_ films were deposited using 4 ALD cycles consisting of alternating exposures of TDMAZ and H_2_O. Each cycle followed a pulse-purge sequence: 2 ×[TDMAZ pulse (50 ms), N_2_ purge (12 s)], 2 × [H_2_O pulse (100 ms), N_2_ purge (12 s)]. Preliminary 1 s H_2_O pulse was applied to ensure nucleation initiation on the bare SiO_2_/Si surface. The reaction chamber temperature was maintained at 150 °C.

The thickness of the deposited ZrO_2_ films was estimated by spectroscopic ellipsometry (J. A. Woollam, Lincoln, NE) using variable incidence angles of 55–75° and a wavelength range of 192–1690 nm. The resulting thickness was approximately 0.5 nm in both cases, corresponding to about one monolayer and confirming good reproducibility across different batches. Ellipsometric data were fitted using a Cauchy model for the Si substrate with a 2 nm native SiO_2_ layer, as determined prior to deposition.

#### 4.1.2 Clusters Deposition

The synthesis of size-selected Cu_*x*_Pd_*y*_ clusters (*x* + *y* = 5), together with pure Pd_5_ and Cu_5_ clusters with atomic precision, was performed in a gas-phase molecular-beam apparatus. The vacuum-based system consists of three interconnected chambers that are differentially pumped with turbomolecular pumps backed by rotary fore-vacuum pumps. Cluster production is achieved using a gas aggregation cluster source for ionic cluster generation, followed by an ion guide and beam optics system for beam collimation and transport and a quadrupole mass spectrometer (QMS) for mass selection.

Charged clusters were generated using a magnetron-based sputtering source of the Haberland type powered by an Advanced Energy MDX 500 DC power supply. The aggregation chamber was cooled with liquid nitrogen. Argon and helium were used as sputtering and carrier gases, respectively, with a typical total gas flow of 300–500 sccm. The magnetron was equipped with a 1 in. diameter Cu–Pd alloy sputtering target (3 mm thick, Cu: Pd ratio 7:3, purity 99.99%; ACI Alloys, USA) for the deposition of bimetallic pentamers and pure palladium clusters. Pure Cu_5_ clusters were produced using a copper target (3 mm thick, purity 99.99%; ACI Alloys, USA).

The beam of positively charged clusters with different sizes was guided downstream through a series of conical and linear octupoles to a quadrupole mass spectrometer (QMS, Extrel, USA), enabling size selection in the mass range of 10–2000 amu. At this stage, the QMS was operated in scanning mode to determine the overall cluster size distribution, while the beam was directed to a detector consisting of a dynode and a channel electron multiplier. By monitoring the resulting mass spectra, the operating parameters of the apparatus were optimized to achieve the highest possible deposition rate for the desired cluster size. Typical mass spectra for the produced bimetallic clusters are shown in Scheme 1.

Subsequently, clusters with the desired compositions were selected by the QMS and directed through a quadrupole bender toward the ZrO_2_ support. The flux of charged clusters landing on the support was measured in real time by using a picoammeter (Keithley 6487), which was also used to bias the substrate and control the impact energy of the clusters during deposition. Soft-landing conditions were maintained with a cluster kinetic energy below 1 eV per metal atom. Using custom Python software, the deposition current was monitored online and integrated over time to determine the total amount of deposited charge. This allowed the number of deposited clusters (and atoms) and the corresponding atomic monolayer equivalent surface coverage (% ML) to be calculated. Typical deposition currents were in the sub-nA range. The deposition spot diameter was approximately 8 mm.

For catalytic testing, size-selected pentamers were deposited on a 0.5 nm thick ZrO_2_ film supported on Si chips with dimensions of 20 × 22 mm^2^. Two deposition spots were prepared, each with a diameter of approximately 8 mm (corresponding to an area of about 0.5 cm^2^ per spot), with a surface coverage of 10% ML. Samples for *ex situ* and *in situ* characterization were prepared on Si chips with dimensions of 8 × 8 mm^2^ and/or 10 × 10 mm^2^, with a single deposition spot at a similar surface coverage of 10% ML. A detailed description of the cluster synthesis procedure can be found elsewhere.^74^

### 4.2 Characterization

#### 4.2.1 Morphology and Structure by AFM and SEM

The topography of the catalysts was characterized by atomic force microscopy (AFM) using a MultiMode 8 microscope (Bruker, USA) operating in tapping mode under ambient conditions. SCANASYST-AIR cantilevers (Bruker, USA) with a resonance frequency of ≈ 75 kHz were used with a nominal tip radius of 2 nm. The processing of AFM images was performed using Gwyddion Software. The statistical information about the surface topography reported in the study was calculated from AFM images of 0.5 μm × 0.5 μm. Further, Mira III scanning electron microscope (Tescan, Czech Republic) operating at an accelerating voltage of 30 kV was used to investigate the morphology of tested catalysts. SEM images were obtained by using a secondary electron detector.

#### 4.2.2 *In Situ* Grazing-Incidence Wide-Angle X-ray Scattering (GIWAXS)

*In situ* GIWAXS measurements were carried out at the SIXS beamline at the SOLEIL synchrotron under UHV conditions, under gas exposure, and at various catalytically relevant temperatures. The diffractometer was equipped with an XPAD140 2D detector from imXPAD company (with an active detector area of 560 × 240 pixels of 130 × 130 μm^2^ in size) for *in situ* surface X-ray scattering data collection. The measurements were performed at a photon energy of 8.8 keV just below the absorption K-edge (to avoid fluorescence) with an incidence angle μ = 0.16° just below the total reflection angle (to enhance the weak metal scattering coming from a low deposited amount of cluster). WAXS patterns were collected in the plane of the substrate by scanning the 2θ scattering angle from 23.5° to 100° with a step size of 0.2° corresponding to a *q* scattering momentum range of 18.2–68.3 nm^–1^ to reveal the structural evolution of the deposited clusters.

Measurements under UHV conditions were conducted on selected samples at fixed temperature over a range from room temperature up to 400 °C with a step of 50–100 °C. The base pressure in the beamline end-station chamber dedicated for UHV surface and interface analysis was approximately between 4 × 10^–10^ and 1 × 10^–9^ mbar.

*In situ* temperature-dependent measurements were performed under a gas atmosphere of cyclohexene and O_2_ with a 1:1 ratio and a total pressure of 1 × 10^–6^ mbar (the maximum pressure allowed by the instrument). The gases were mixed in a custom-built gas handling system using two EVR116 regulating valves (Pfeiffer, Germany) and RVC300 control units (Pfeiffer, Germany). The mixing pressure was approximately 1 mbar, measured with a Pfeiffer PKR251 full-range gauge, and the gas mixture was subsequently introduced into the measurement chamber through a needle valve to reach the target pressure.

#### 4.2.3 Near-Ambient-Pressure X-ray Photoelectron Spectroscopy (NAP-XPS)

A laboratory-based near-ambient-pressure X-ray photoelectron spectroscopy (NAP-XPS) system (SPECS Surface Nano Analysis GmbH) was used to evaluate the surface chemical composition of the catalysts. The instrument was equipped with a monochromatized Al Kα (*h*ν = 1486.6 eV) X-ray source and a hemispherical electron energy analyzer with a 1D-DLD multichannel detector (SPECS Phoibos 150). During measurements under ultrahigh vacuum (UHV) conditions, the pressure in the analysis chamber was about 2 × 10^–9^ mbar. Survey spectra were acquired in the binding energy (BE) range of −2 to 1100 eV using a pass energy of 50 eV. High-resolution spectra were recorded with a pass energy of 20 eV. Depending on the intensity of the measured region, the average of 10–80 scans was used to obtain spectra with a low noise-to-signal ratio.

*In situ* measurements under reaction conditions were performed using a mixture of cyclohexene and oxygen (C_6_H_10_:O_2_, ratio 1:1) at a total pressure of 1 mbar. For these measurements, a dedicated set of bimetallic Cu_*x*_Pd_*y*_ pentamers, as well as pristine Pd_5_ and Cu_5_ clusters, supported on ZrO_2_ films, was prepared on n-type Si chips (10 mm × 10 mm) to provide sufficient electrical conductivity and minimize charging effects. The cluster coverage was kept at 10% ML, identical to that used in the catalytic tests. Measurements under reducing and oxidizing environments were carried out using pure C_6_H_10_ and pure O_2_, respectively, with pressures maintained at approximately 0.5 and 0.9 mbar.

#### 4.2.4 XPS Data Analysis

XPS spectra were analyzed using CasaXPS software. All binding energies used for component analysis were referenced to the Si 2p_3/2_ core level of the Si–Si bond, set at 99.2 eV, corresponding to a Si 2p peak position of 99.4 eV.^52^ In all cases, background subtraction was performed using a Shirley background. Core-level spectra were fitted using the symmetric LA­(1.53,243) line shape, which represents a Voigt-type profile (a convolution of Gaussian and Lorentzian distributions) and is the default option for symmetric fitting in CasaXPS. For the Pd 3d region, an LA­(1,643) line shape was used to obtain the best fit. For certain spectral regions (Si 2p), the line shape parameters were slightly adjusted to minimize the standard deviation of the residuals.

The characteristic spin–orbit splitting between Pd 3d_5/2_ and Pd 3d_3/2_ peaks was allowed to vary freely, and remained in the range reported in the literature (5.3 ± 0.12 eV). The area of each Pd 3d_3/2_ component was constrained to 66.67% of the corresponding Pd 3d_5/2_ component, maintaining the expected 2:3 area ratio for the spin–orbit doublet. Because the Cu 2p region exhibits a large spin–orbit splitting (Δ = 19.75 eV), only the Cu 2p_3/2_ region was measured. The Cu LMM Auger line was not recorded due to the very low signal intensity resulting from the small cluster coverage and the limited measurement time during the NAP-XPS beamtime.

For most elements, the full width at half-maximum (fwhm) was below 2 eV and was generally allowed to vary during the fitting procedure. Constraints were applied only to define a maximum allowed fwhm in cases where multiple fitting components were required to obtain a stable fit. An exception was made for Cu­(II) components and their associated shakeup satellite peaks, which exhibit significantly broader features. In these cases, the fwhm was constrained to a maximum of 4 eV when necessary.

The assignment of components during deconvolution of the spectra was based on standard references 44,49 and on literature reports for size-selected clusters, taking into account intrinsic binding energy shifts associated with the size effects of small supported clusters, as discussed in the main text. The Pd 3d spectral region partially overlaps with the Zr 3p signal; however, this overlap does not influence the analysis or interpretation of the spectra. Because all samples were exposed to ambient air prior to measurements, a significant amount of carbonaceous species was present on the surface (approximately 20 atom %), which decreased to about 12 atom % at elevated temperatures. All NAP-XPS spectra were normalized to their maximum intensity for a better comparison.

### 4.3 Catalytic Testing

Temperature-programmed reaction (TPR) was used to determine the performance of the catalysts. The setup consists of the reaction cell, mass spectrometer system, gas mixer, and combination of electric valves, control units, and pumps to operate in constant pressure conditions during the heating and cooling temperature ramps for hours. The reaction cell is custom-built from aluminum alloy and represents a fixed-bed continuous flow catalysis reactor with an internal volume of approximately 33 cm^3^ equipped with a boron nitride-covered pyrolytic graphite heater. The body of the cell was water-cooled to 22 °C. The pressure inside the cell was maintained at 800 Torr by implementing a regulation loop utilizing a downstream mass-flow controller (SLA5850, Brooks, USA) connected to the diaphragm pump (Divac 1.4HV3, Pfeiffer, Germany) and a pressure transducer (PX209, Omega, USA) so that the readout from the pressure transducer was monitored by a custom software (written in Python) and the flow through the downstream controller was regulated accordingly to maintain the preset pressure of 800 Torr. The reactant gases were introduced using a gas mixer (Swagelok) equipped with the assembly of mass-flow controllers (Brooks SLA5850). The total gas flow through the cell was kept constant at 17.5 sccm. Prior to the measurements, the setup was evacuated by using a membrane pump and flushed with helium flow. The purging cycle was repeated five times, whereupon the reaction cell flushed continuously for 2 h with pure helium (10 sccm). After the introduction of the reactants, the temperature ramp was started after 4 h, when a steady signal intensity was obtained in the mass spectrometer for the cyclohexene and oxygen reactants. Reaction products were monitored using a differentially pumped mass spectrometer Prisma Plus (Pfeiffer, Germany) with a mass range *m*/*z* of 1–100 amu.

Three reaction conditions were examined using C_6_H_10_:O_2_ feed ratios of 1:1 (stoichiometric ODH), 1:0.1 (oxygen-deficient or substoichiometric ODH), and 1:0 (nonoxidative dehydrogenation, DH). Each TPR experiment consisted of a programmed heating ramp from room temperature (RT) to 300 °C followed by cooling to 25 °C, with measurements recorded at 50 °C intervals starting from 100 °C and ramping rate of 10 °C/min. For the 1:1 feed, two consecutive cycles were performed; only the second cycle is discussed, as the first ramp is typically influenced by residual carbonaceous species, leading to their removal and to catalyst surface transformation or activation. The first temperature ramp in fact acts as a pretreatment/activation phase for all catalysts. Hence, one measurement included 4 test ramps running one after another. The dwelling time at each temperature was set to 120 min to ensure a steady/stabilized signal during mass spectra acquisition.

The acquired data were then processed as follows: time-dependent intensity correction (all product intensities were normalized to the maximum intensity of cyclohexene peak) and background correction (for each monitored *m*/*z*, the signal measured for a blank ZrO_2_-on-Si sample, which showed no detectable catalytic activity, was subtracted from the corresponding signal measured for the catalyst sample). The reaction rates for the individual products were obtained by taking into account the sensitivity of the mass spectrometer to calibrated gas mixtures and normalizing them to the total surface area of catalysts. Product rates were calculated as the number of molecules per unit time (s) per atom in the cluster. Additionally, Pd-normalized rates were provided in Figure S2. To quantify the distribution of hydrogen-containing products under ODH conditions (H_2_/H_2_O ratios in Figure 1c), the selectivities toward molecular hydrogen and water were calculated according to

$$s_{H_{2}}=\frac{r_{H_{2}}}{r_{H_{2}}+r_{H_{2}O}}\times 100\%$$

and

$$s_{H_{2}O}=\frac{r_{H_{2}O}}{r_{H_{2}}+r_{H_{2}O}}\times 100\%,$$

where *s*_H_2__ and *s*_H_2_O_ denote the selectivities toward H_2_ and H_2_O, respectively, and *r*_H_2__and *r*_H_2_O_ represent the corresponding formation rates. Detailed information on the TPR design and data analysis procedure can be found in our previous works.^35,75^

Kinetic measurements were performed using the Cu_3_Pd_2_/ZrO_2_ catalyst with a cluster coverage of 10% ML, selected because of its high catalytic activity and good stability under the investigated reaction conditions. The first two temperature ramps (R1 and R2) were performed under the standard feed conditions to reproduce the catalytic testing protocol. The standard feed composition of 0.30% C_6_H_10_ and 0.30% O_2_ (ratio: 1:1) was subsequently repeated throughout the extended kinetic sequence (R3, R5, R9, and R13) to monitor catalyst stability and account for gradual changes in catalytic activity. For determination of the apparent cyclohexene reaction order, the C_6_H_10_ concentration was varied from 0.15 to 0.30% (0.15, 0.20, 0.25, and 0.30%), corresponding to partial pressures of 145.3, 193.7, 239.8, and 288.2 Pa, respectively, while the O_2_ concentration was maintained at 0.30% (288.9 Pa). For determination of the apparent O_2_ reaction order, the O_2_ concentration was varied from 0.20 to 0.30% (0.20, 0.25, and 0.30%), corresponding to partial pressures of 190.7, 242.7, and 288.9 Pa, respectively, while the C_6_H_10_ concentration was maintained at 0.30% (288.2 Pa). For each gas composition, a complete temperature series was recorded at 270, 280, 290, and 300 °C, while the total pressure and total gas flow were maintained constant. Benzene formation rates and the corresponding H_2_/H_2_O product distributions were determined from calibrated mass-spectrometric signals and used for reaction-order, Arrhenius, and hydrogen-product-branching analyses. The H_2_ fraction was calculated as

$$\frac{r_{H_{2}}}{r_{H_{2}}+r_{H_{2}O}}.$$

The subsequent 24 h stability test at 290 °C was performed under standard reaction conditions.

### 4.4 Density Functional Theory Calculations

Periodic density functional theory (DFT) calculations were conducted utilizing the Vienna Ab Initio Simulation Package (VASP).^76,77^ The interactions between atomic cores and valence electrons were described by the projector-augmented wave (PAW) method.^78^ The valence electron configurations explicitly accounted for in this study include H (1s^1^), C (2s^2^, 2p^2^), O (2s^2^, 2p^4^), Cu (3d^10^, 4s^1^), Pd (4d^9^, 5s^1^), and Zr (4s^2^, 4p^6^, 4d^2^, 5s^2^). To treat electronic exchange and correlation, the generalized gradient approximation (GGA) in the Perdew–Burke–Ernzerhof (PBE) formulation was employed.^79^ To accurately represent long-range dispersion forces, which are critical for the adsorption of cyclic hydrocarbons, the DFT-D3 method with Becke–Johnson (BJ) damping was applied.^80^ For a more precise description of the electronic structure of the ZrO_2_ support, an on-site Coulomb interaction correction (DFT+*U*) was applied to the Zr 4d electrons according to the Dudarev approach.^81^ An effective Hubbard parameter (*U*_eff_) of 4 eV was utilized,^82^ ensuring an accurate qualitative representation of the structural and electronic properties of zirconium oxide.

A kinetic energy cutoff of 500 eV was established for the plane-wave basis set. Brillouin zone sampling was conducted with a reciprocal space distance of 0.04 Å^–1^, yielding a (7 × 7 × 5) k-point grid for bulk tetragonal ZrO_2_ (t-ZrO_2_) calculations. For the slab, Γ point-only sampling was used. Electronic and ionic relaxations were considered converged when residual changes in total energy fell below 10^–5^ eV and 10^–4^ eV, respectively. This theoretical framework allows for the accurate determination of key electronic descriptors, such as d-band centers and Bader charges, which are used to interpret the composition-dependent catalytic performance.

### 4.5 Machine-Learning Interatomic Potentials

#### 4.5.1 Gaussian Approximation Potentials

The interatomic potential for the Zr–O–H system was parametrized within the Gaussian approximation potential (GAP) framework,^83^ utilizing a combination of two-body and many-body descriptors to represent the potential energy surface. Specifically, two-body interactions were modeled using a distance-based kernel with a cutoff of 6.0 Å and 10 sparse points, employing an automatic relevance determination (ARD) squared exponential covariance type.

To capture higher-order many-body interactions across multiple length scales, we implemented a “double turbo-SOAP” (smooth overlap of atomic positions) approach.^84,85^ This involved assigning two turbo-SOAP kernels to each chemical species (Zr, O, and H) with hard cutoffs (*r*_cut,hard_) of 4.0 Å and 6.0 Å. These descriptors were expanded using *l*_max_ = 4 and *n*_max_ = 8 with a poly3gauss basis set to ensure a smooth transition at the cutoff boundaries. To maintain a sparse yet representative mapping of the local atomic environments, 4,000 sparse points were selected for each kernel via the CUR points method. The training process was regularized by assigning standard deviations (σ) of 0.001 eV for total energies, 0.1 eV/Å for forces, and 0.05 eV for virials, thereby ensuring a balanced fit across diverse physical properties of the training set.

To ensure the robustness and transferability of the GAP, particularly for the amorphous ZrO_2_ surface and its complex interactions with hydrogen, we implemented an active learning-based iterative refinement protocol.^86,87^ During molecular dynamics (MD) simulations performed in LAMMPS,^88^ the local Gaussian uncertainty of the sampled configurations was systematically monitored. Structures exhibiting a local uncertainty of ≥ 50 meV/atom were extracted as candidates for the training set. To maintain a diverse and nonredundant training set, a similarity analysis was subsequently conducted;^87^ the configuration with the maximum uncertainty served as a reference, while structurally similar configurations within the uncertain pool were discarded. These selected high-uncertainty structures were then subjected to high-level DFT calculations to obtain reference energies, forces, and virials. This refinement cycle was iterated until the structures sampled during the MD simulations no longer displayed significant uncertainty or the prediction error between the machine-learning interatomic potential (MLIP) and the DFT reference values reached our target convergence threshold (20 meV/atom for energy and 0.25 eV/Å for forces).

#### 4.5.2 MACE Potentials

For the complex bimetallic cluster–support interface involving Cu, Pd, Zr, O, C, and H, we utilized the MACE framework.^89,90^ MACE was selected due to its superior ability to handle high-dimensional elemental embeddings, which is essential as the chemical complexity of the system increases beyond the capabilities of the GAP model used for the bare support. To accelerate the convergence and enhance the robustness of the potential, we adopted a transfer learning approach using a pretrained foundation model (mace-mpa-0-medium).^91^ This model was subsequently fine-tuned using a multihead architecture, allowing for the precise capture of the potential energy surface across diverse chemical environments.

The MACE models were trained with a many-body cutoff (*r*_max_) of 6.0 Å with a learning rate of 0.005. To ensure numerical stability and prevent unphysical atomic overlaps during global optimization, a pair repulsion term was incorporated. We employed stochastic weight averaging (SWA) and exponential moving average (EMA) with a decay rate of 0.995 to enhance the generalization performance and stability of the potential. All calculations were performed in double precision to maintain high-level accuracy for energy and force predictions, with loss function weights assigned as 1.0 for energies and 10.0 for forces.

The development of the MACE potential was facilitated by a hierarchical active learning strategy that systematically expanded the training set to encompass the complexity of the catalytic interface.^92^ This refinement process began with the modeling of Cu–Pd pentamer adsorption on the amorphous ZrO_2_ surface, incorporating diverse surface hydroxylation states to accurately represent the experimental support conditions. Once the stable cluster-support motifs were established, the potential was further augmented to include the adsorption of cyclohexene (C_6_H_10_), providing a rigorous description of the reactant’s interaction with the clusters.

To systematically refine the training set, we implemented a query-by-committee (QbC) approach.^93^ An ensemble of three MACE models was trained with different initial seeds, and the standard deviation of the atomic forces among the committee members was used as a metric for neural network (NN) uncertainty. During the structural exploration via basin hopping, configurations exhibiting a maximum force uncertainty of ≥ 0.2 eV/Å were flagged. To ensure the physical validity of the newly selected data, we applied filtering criteria: structures were only submitted for DFT validation if their maximum atomic force was below 30 eV/Å and they passed a connectivity analysis to confirm structural integrity. This iterative process ensured that the potential remained accurate in the most catalytically relevant regions of the configuration space.

### 4.6 Generation of the Amorphous ZrO_2_ Support

The amorphous ZrO_2_ support models were generated using a modified melt-cleave-quench approach.^94^ The initial configuration was derived from bulk t-ZrO_2_, which was reoriented along the (101) crystallographic plane. This oriented structure was subsequently expanded into a (5 × 3 × 2) supercell to provide a sufficiently large atomistic domain for the disordered phase.

The atomistic simulations were performed using the LAMMPS molecular dynamics package, using GAP. To eliminate any crystalline memory, the ZrO_2_ supercell was first subjected to a randomization phase at 6000 K within the NVT ensemble for 10 ps, ensuring a fully disordered liquid state. Following randomization, the system was transformed into a slab geometry by expanding the simulation cell along the *z*-axis. A vacuum layer of 20 Å was introduced on the molten oxide to create a liquid slab. The resulting liquid ZrO_2_ slab was then equilibrated at 4000 K for 100 ps. During this high-temperature stage, harmonic walls were applied at the *z*-boundaries to stabilize the liquid interfaces while maintaining the slab morphology. The amorphous solid was obtained through a systematic quenching procedure from 4000 to 300 K. To investigate the influence of thermal history on the support properties, three distinct linear cooling rates were employed: 10 K/ps, 25 K/ps, and 100 K/ps. After reaching 300 K, the slabs were equilibrated for an additional 10 ps to relax the disordered network. Finally, the structures underwent a rigorous energy minimization using the conjugate gradient algorithm to obtain the representative amorphous ZrO_2_ surfaces used for subsequent cluster deposition and catalytic modeling.

### 4.7 Global Optimization and Ensemble Sampling

#### 4.7.1 Canonical Basin Hopping for Bare Clusters

To identify the most stable configurations and thermally accessible isomers of the bimetallic Cu_*x*_Pd_*y*_ pentamers on the amorphous ZrO_2_ support, global optimization was performed by using a canonical basin hopping (BH) approach. This was implemented through an in-house global optimization code previously developed for subnanometer cluster systems.^63^ In this initial stage, the search was conducted within a canonical ensemble, where the number and composition of the metal atoms in the pentamers were kept constant to isolate the intrinsic cluster-support interactions from environmental effects.

The global optimization was driven by the MACE MLIP, integrated with the atomic simulation environment (ASE) through a customized Python framework.^95^ The search algorithm systematically explored the complex potential energy surface (PES) by employing a variety of stochastic trial moves including random atomic perturbations, cluster displacements, and rotations. Each trial configuration was subjected to a rigorous local geometry optimization using the MACE-ASE calculator to reach the nearest local minimum. A Metropolis criterion was then applied to accept or reject the newly optimized structures, facilitating the transition across energy barriers and enabling the identification of the global minimum and low-energy metastable isomers. This canonical sampling provides a foundational library of bare cluster geometries, which serves as the starting point for subsequent simulations under reactive environments.

#### 4.7.2 Grand Canonical Basin Hopping (GCBH) for Reactive Environments

To accurately simulate the catalytic interface under realistic reaction conditions, grand canonical basin hopping (GCBH) simulations were performed starting from the stable bare cluster configurations identified in the previous stage. In this section, the experimental environment within the reaction cell at 300 °C was explicitly accounted for by utilizing the partial pressures of oxygen (O_2_) and water vapor (H_2_O). These experimental partial pressures were converted into atomic chemical potentials (μ_O_ and μ_H_) using statistical mechanical relations, which served as the primary input parameters for the GCBH algorithm.

Unlike the canonical approach, the GCBH algorithm enables the system to optimize its oxidation state and degree of surface hydroxylation by allowing for the stochastic adsorption and desorption of oxygen and hydrogen atoms. The simulation incorporated various grand canonical moves, including add_O, remove_O, add_H, remove_H, and add_OH, with the acceptance of each trial move determined by the Metropolis criterion based on the computed grand potential. Notably, for the Cu_5_ cluster, which exhibited no experimental reactivity, the μ_H_ was omitted. In this case, only μ_O_ was utilized to focus on the oxidation of the cluster under the reactive environment. For all other active cluster compositions, both μ_O_ and μ_H_ were applied to sample a comprehensive ensemble of partially oxidized and hydroxylated interfaces, reflecting the dynamic equilibrium between the subnanometer catalysts and the gas phase.

#### 4.7.3 Boltzmann-Populated Ensemble Analysis

The representative stable ensemble for each cluster composition was determined by performing a statistical thermodynamic analysis of the configurations sampled during the GCBH trajectories. Because the GCBH process samples states with varying atomic populations, the relative stability of each configuration was evaluated using the grand potential (Ω), calculated as the total energy predicted by the MACE potential minus the sum of the products of the atom counts and their respective atomic chemical potentials (μ_*i*_) for all constituent species, including the ZrO_2_ support, bimetallic constituents (Cu, Pd), and reactive species (O, H).

The statistical weight of each state within the accessible configurational space was determined by applying Boltzmann statistics at the experimental reaction temperature of 300 °C. The Boltzmann probability (*P*_*i*_) for each configuration was calculated as

$$P_{i}=\frac{\exp\left[-\Delta \Omega_{i}/(k_{B}T)\right]}{\underset{j}{\sum }\exp\left[-\Delta \Omega_{j}/(k_{B}T)\right]},$$

where ΔΩ_*i*_ is the grand potential of configuration *i* relative to the global minimum identified for that specific composition. To filter the vast configurational space into a representative set of catalysts, a selection criterion was applied to include only configurations with a Boltzmann probability of 1% or higher. These thermally relevant structures were then sorted by their statistical weight and utilized for subsequent high-level DFT refinements to extract the electronic and geometric descriptors, such as d-band centers and Bader charges, used to interpret the catalytic performance.

## Supplementary Material



## Data Availability

The data that support the findings of this manuscript are available from the corresponding authors upon reasonable request.

## Notes

Time-resolved evolution of reactants for all temperature ramps; Pd-normalized rates; kinetic analysis of benzene formation over Cu_3_Pd_2_/ZrO_2_ and related H_2_/H_2_O branching; evolution of the GIWAXS diffraction patterns; high-resolution Zr 3d, Cu 2p_3/2_ and Pd 3d spectra for all pentamer catalysts measured by NAP-XPS; DFT ensemble configurations; overlay of experimental NAP-XPS and ensemble-weighted theoretical Cu 2p_3/2_ spectra with description; table of gas-phase partial pressures used for the theoretical calculations; free-energy calculations for the first O-assisted C–H activation; table of electronic and free-energy results for the six first O-assisted C–H activation pathways; combined MLIP-level NEB profiles for the six first O-assisted H-transfer pathways (PDF)
