# Supplementary material for: Oxygen-Promoted Dehydrogenation on Size-Selected Cu–Pd Pentamer Clusters for Low-Temperature Hydrogen Release from Cyclic Hydrocarbons
Source: ACS Nano. 2026 Oct 27;20(44):29810–32. doi: 10.1021/acsnano.6c12121 (PMC13758396; doi:10.1021/acsnano.6c12121)
Supplement: Supplementary file 1 [file nn6c12121_si_001.pdf]

Supporting Information

Oxygen-Promoted Dehydrogenation on Size-Selected Cu–Pd Pentamer Clusters for Low-Temperature Hydrogen Release from Cyclic Hydrocarbons

Mykhailo Vaidulych[1§*], Yonghyuk Lee[2,3§], Federico Loi[1], Petr Vitek[1], Michael Vorochta[4], Stanislav Valtera[1], Ivan Khalakhan[4], Claudia de Melo[5], Samiran Chakraborty[4], Karolína Simkovičová[1], Yves Garreau[6], Benjamin Voisin[6], Bilal Bawab[7,8], Jan M. Macak[7,8], Muntaseer Bunian[9], Moon-Hyung Jang[9], Yu Lei[9], Philippe Sautet[2], Iva Matolinová[4], Alessandro Coati[6], Pascal Andreazza[5], Anastassia N. Alexandrova[2*], Stefan Vajda[1*]

[§]Mykhailo Vaidulych and Yonghyuk Lee contributed equally and has been treated as co-first authors to this study.
[*]Corresponding authors: Mykhailo Vaidulych (mykhailo.vaidulych@jh-inst.cas.cz), Anastassia Alexandrova (alexandrova@g.ucla.edu) and Stefan Vajda (stefan.vajda@jh-inst.cas.cz)

[1] Department of Nanocatalysis, Heyrovský Institute, Czech Academy of Sciences, Dolejškova 2155/3, 18200 Prague 8, Czech Republic
[2] Department of Chemistry and Biochemistry, University of California, Los Angeles, California 90095, United States
[3] Department of Chemical and Biochemical Engineering, Dongguk University, Seoul 04620, Republic of Korea
[4] Department of Surface and Plasma Science, Faculty of Mathematics and Physics, Charles University, V Holešovičkách 2,180 00, Prague, Czech Republic
[5] Université d'Orléans, CNRS, ICMN UMR7374, 1b rue de la Férollerie, 45071 Orléans, France.
[6] Synchrotron SOLEIL, L'Orme des Merisiers, Départementale 128, 91190 Saint-Aubin, France
[7] Center of Materials and Nanotechnologies, Faculty of Chemical Technology, University of Pardubice, Pardubice 53002, Czech Republic
[8] Central European Institute of Technology, Brno University of Technology, Purkynova 123, 612 00 Brno, Czech Republic
[9] Department of Chemical and Materials Engineering, University of Alabama in Huntsville, 301 Sparkman Drive, Huntsville, AL 35899, United States

Contents of the Supporting Information

Figure S1. Time-resolved evolution of reactants (cyclohexene and water) and products (benzene, hydrogen, and water) acquired during four consecutive temperature ramps.

Figure S2. Pd-normalized rates of benzene and hydrogen formation.

Figure S3. Kinetic analysis of benzene formation over Cu_3Pd_2/ZrO_2 as a function of reactant partial pressure.

Figure S4. Temperature- and feed-composition-dependent kinetic behavior and H_2/H_2O branching over Cu_3Pd_2/ZrO_2.

Figure S5. Time-dependent evolution of benzene and hydrogen signals for the Cu_3Pd_2 catalyst during a 24 hours stability test under standard reaction conditions.

Figure S6. Evolution of the GIWAXS diffraction patterns as a function of temperature under gas exposure and UHV conditions for all measured catalysts.

Figure S7. High-resolution Zr 3d spectra for all pentamer catalysts measured by NAP-XPS under different temperatures and *in situ* gas atmospheres.

Figure S8. High-resolution Cu $2p_{3/2}$ spectra for all pentamer catalysts measured by NAP-XPS under different temperatures and *in situ* gas atmospheres.

Figure S9. High-resolution Pd 3d spectra for all pentamer catalysts measured by NAP-XPS under different temperatures and *in situ* gas atmospheres.

Figure S10. All possible ensemble configurations of Cu_xPd_y pentamers on ZrO_2 under ODH conditions at 300 °C.

Figure S11. Overlay of experimental NAP-XPS and ensemble-weighted theoretical Cu $2p_{3/2}$ spectra for Cu_2Pd_3, Cu_3Pd_2, Cu_4Pd_1, and Cu_5 obtained/calculated under NAP-XPS reaction conditions at 300 °C.

Section S1. Analysis of the agreement between experimental NAP-XPS and ensemble-weighted theoretical Cu $2p_{3/2}$ spectra (Figure S11).

Table S1. Gas-phase partial pressures used for the theoretical calculations under NAP-XPS-matched and TPR-derived environments at 300 °C.

Section S2. Free-energy calculations for the first O-assisted C-H activation.

Table S2. Electronic and free-energy results for the six first O-assisted C-H activation pathways.

Figure S12. Combined MLIP-level NEB profiles for the six first O-assisted H-transfer pathways.

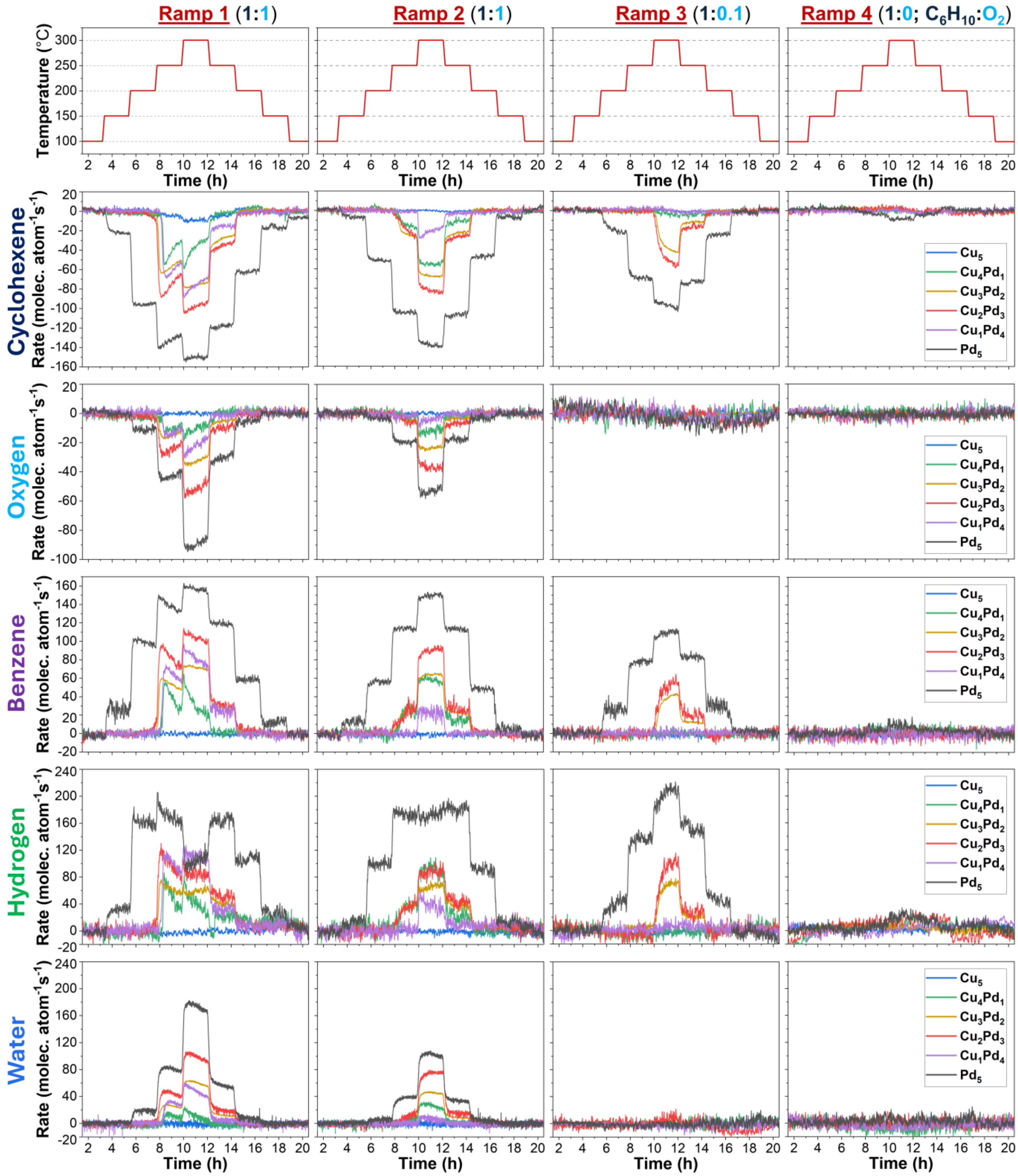

Figure S1. Time-dependent evolution of reactants (cyclohexene and oxygen) and products (benzene, hydrogen, and water) acquired during four consecutive temperature ramps and measured under DH conditions with C_6H_{10}:O_2 ratios of 1:1 (ramps 1 and 2), 1:0.1 (ramp 3), and 1:0 (ramp 4). The data are presented as raw mass spectrometry signals, corrected for baseline back-tilting observed during extended (more than 80 h) measurements and for the background signal from a blank ZrO_2 support (which showed no detectable activity).

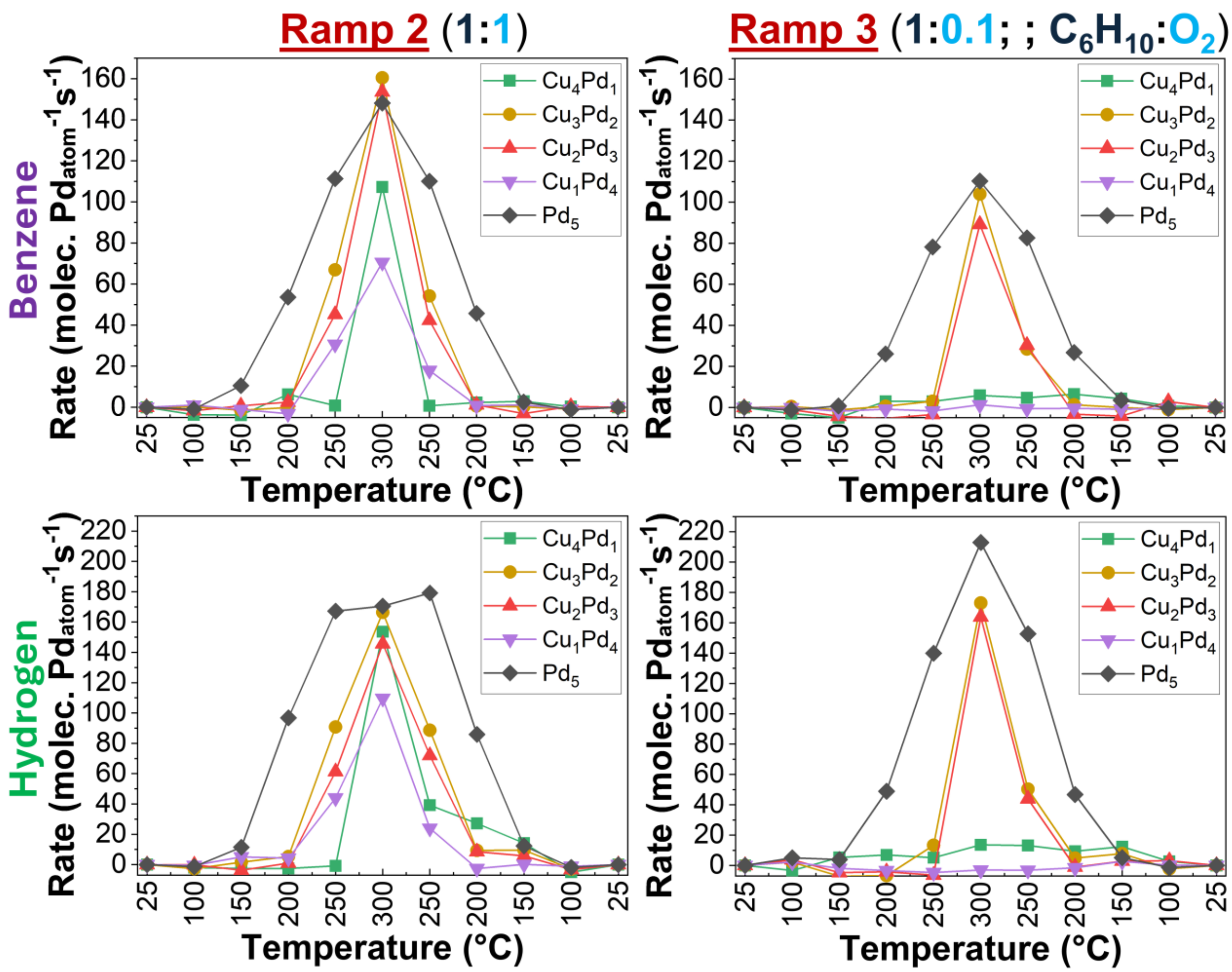

Figure S2. Pd-normalized rates of benzene (top) and hydrogen (bottom) formation during ramp 2 and ramp 3 of the DH reaction for selected samples, with C_6H_{10}:O_2 ratios of 1:1 and 1:0.1, respectively.

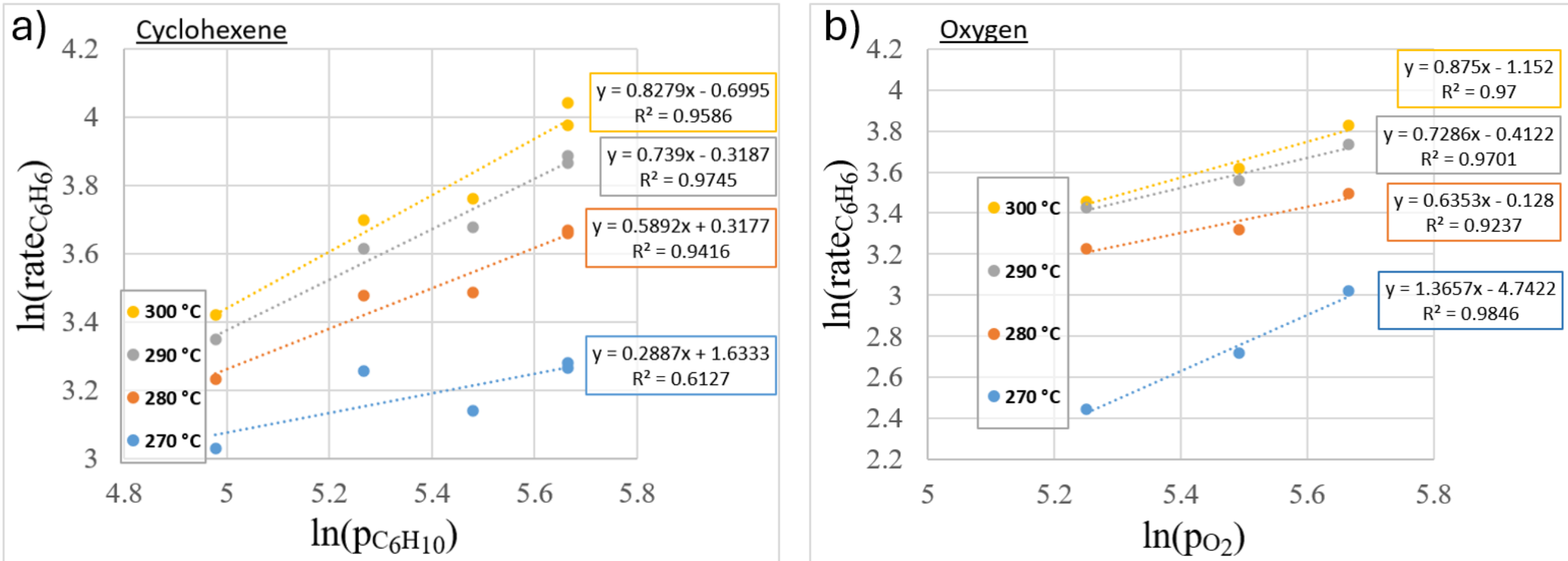

Figure S3. Kinetic analysis of benzene formation over Cu_3Pd_2/ZrO_2 as a function of reactant partial pressure. (a) Dependence of ln(rate$_{C_6H_6}$) on ln(p$_{C_6H_{10}}$) at constant O_2 pressure and (b) dependence of ln(rate$_{C_6H_6}$) on ln(p$_{O_2}$) at constant C_6H_{10} pressure. Measurements were performed between 270 and 300 °C. Linear regression of the log-log rate dependences was used to obtain the corresponding apparent reaction orders in C_6H_{10} and O_2.

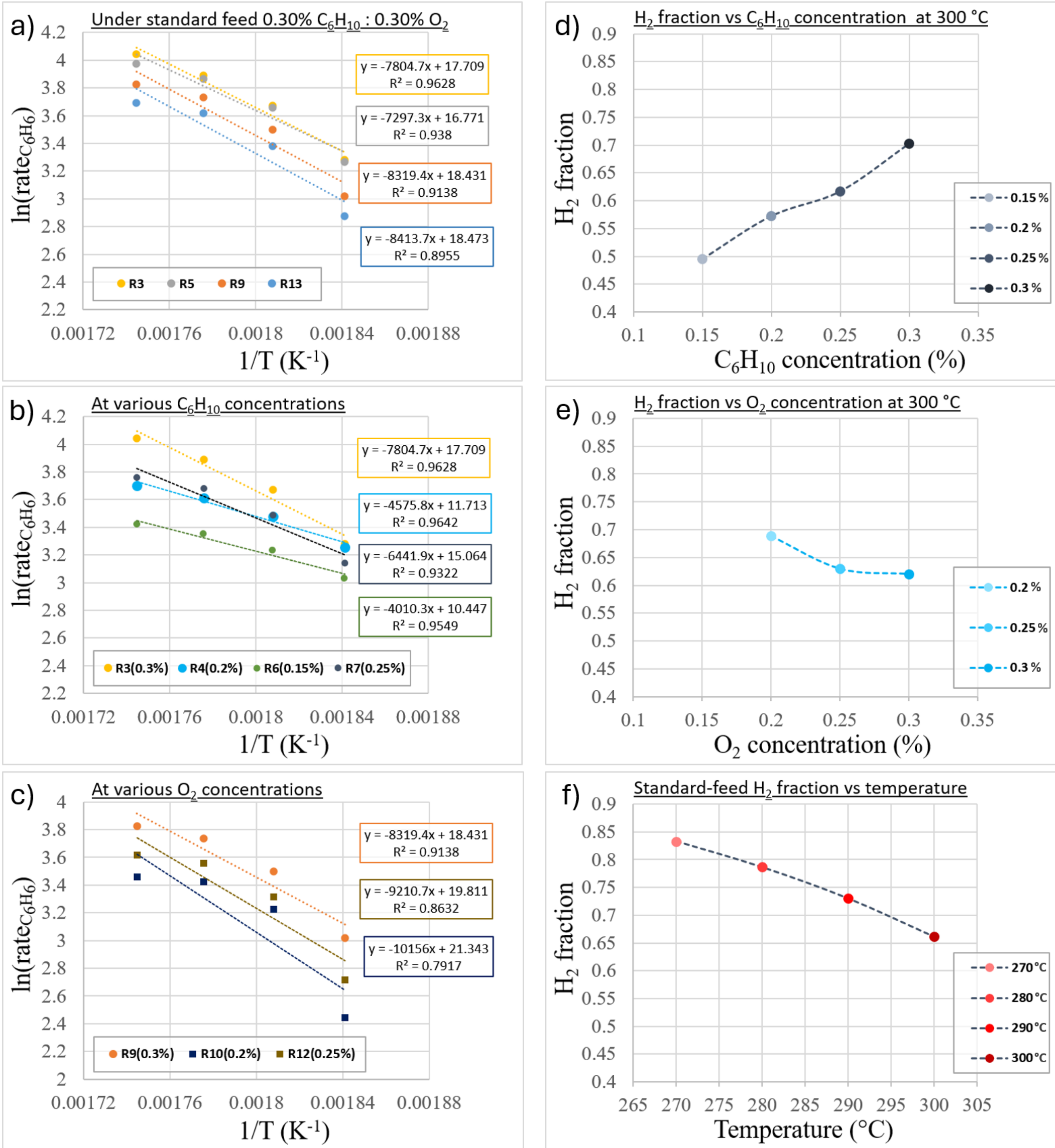

Figure S4. Temperature- and feed-composition-dependent kinetic behavior and H_2/H_2O branching over Cu_3Pd_2/ZrO_2. (a–c) Arrhenius plots of ln($rate_{C_6H_6}$) as a function of 1/T: (a) under the standard feed of 0.3% C_6H_{10} and 0.3% O_2 (C_6H_{10} : O_2 = 1:1) during repeated temperature ramps (R3, R5, R9, and R13) performed throughout the kinetic study; (b) at different C_6H_{10} concentrations and constant O_2 concentration; and (c) at different O_2 concentrations and constant C_6H_{10} concentration. (d–f) H_2 fraction as a function of (d) C_6H_{10} concentration at 300 °C and constant O_2 concentration of 0.3%, (e) O_2 concentration at 300 °C and constant C_6H_{10} concentration of 0.3%, and (f) temperature under the standard feed.

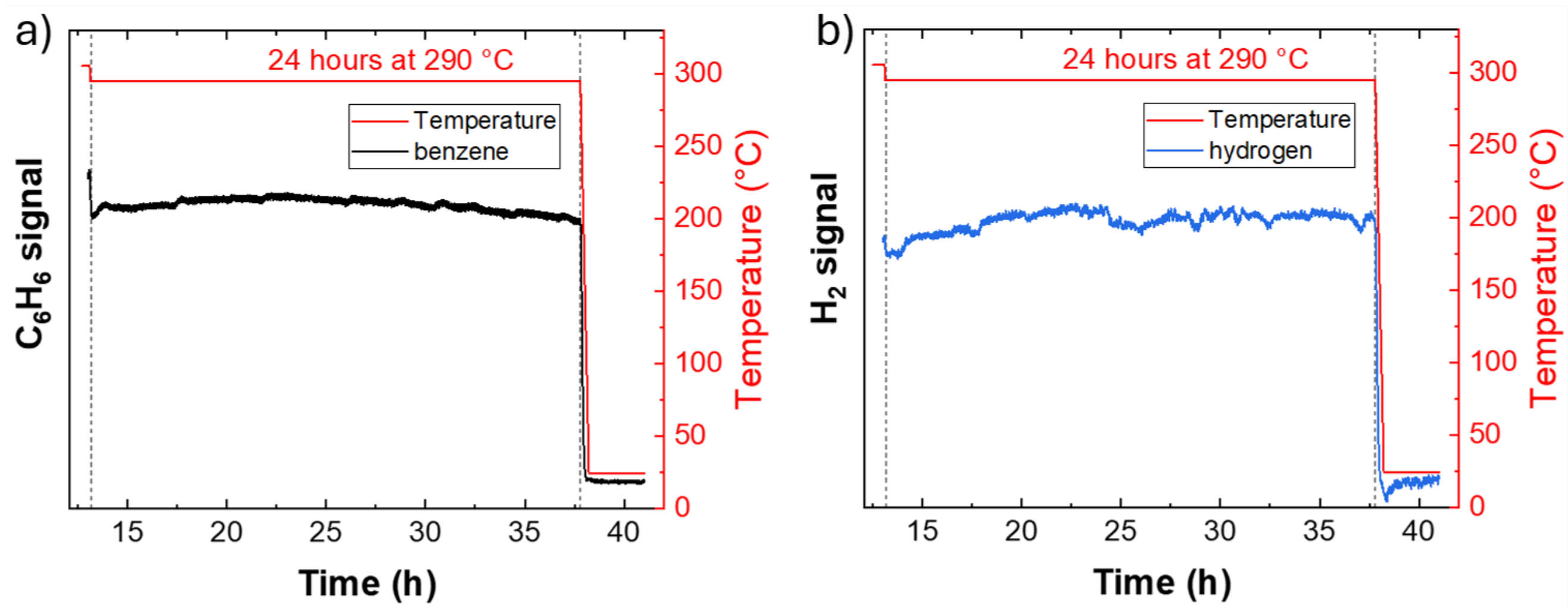

Figure S5. Time-dependent evolution of benzene and hydrogen signals for the Cu_3Pd_2 catalyst during a 24 hours stability test under standard reaction conditions (C_6H_{10}:O_2 = 1:1). The data are presented as raw mass spectrometric signals. The stability test was performed using the same catalyst sample previously subjected to approximately 11 days of kinetic measurements involving repeated temperature ramps and changes in reactant feed composition.

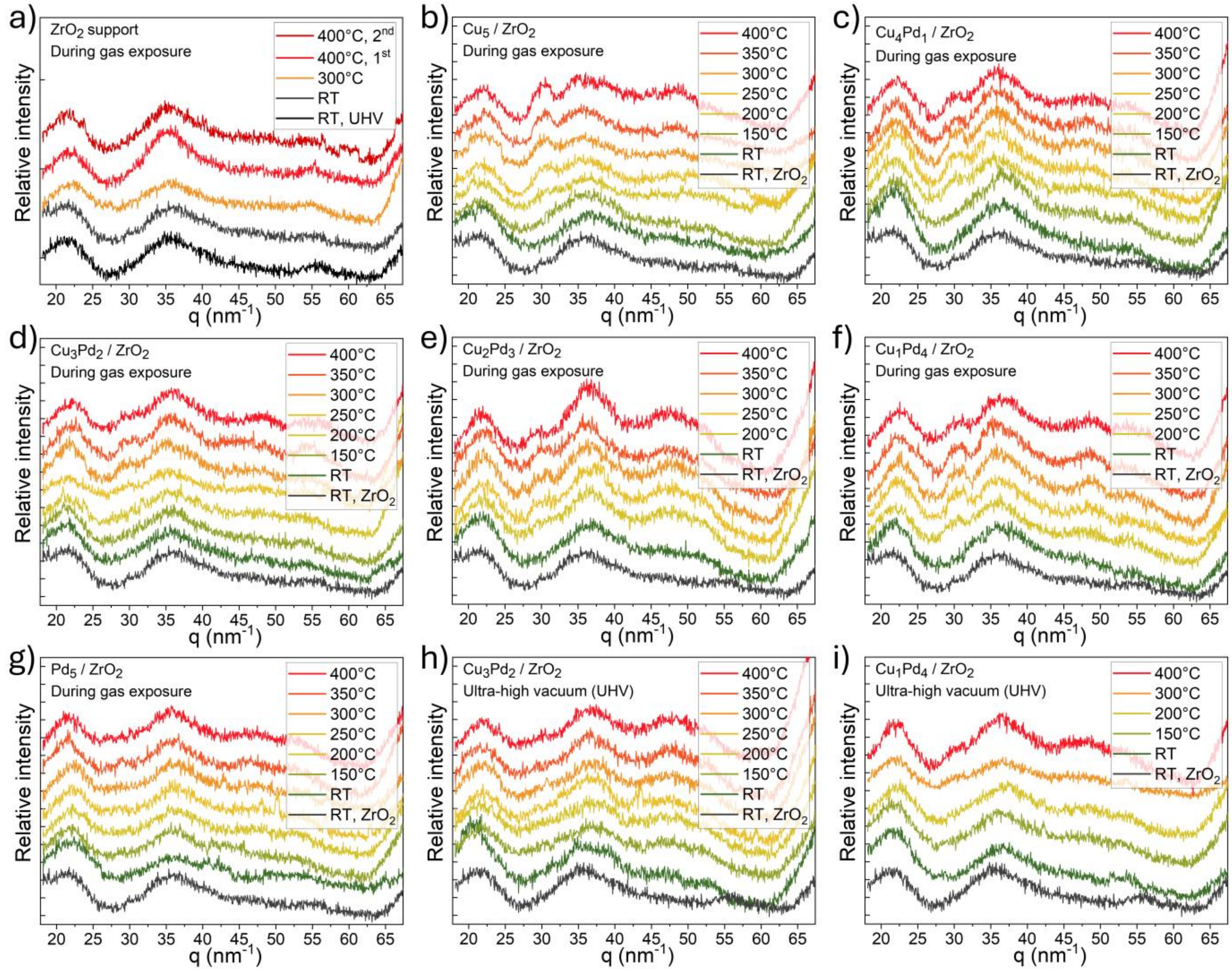

Figure S6. (a–g) Evolution of the GIWAXS diffraction patterns as a function of temperature (up to 400 °C) and gas exposure (C_6H_{10}:O_2 ratio of 1:1) in the 10^{-6} mbar pressure range for the bare ZrO_2 support, Cu_5/ZrO_2, Cu_4Pd_1/ZrO_2, Cu_3Pd_2/ZrO_2, Cu_2Pd_3/ZrO_2, Cu_1Pd_4/ZrO_2, and Pd_5/ZrO_2, respectively. (h, i) Representative GIWAXS patterns for Cu_3Pd_2/ZrO_2 and Cu_1Pd_4/ZrO_2 measured at different temperatures under UHV conditions.

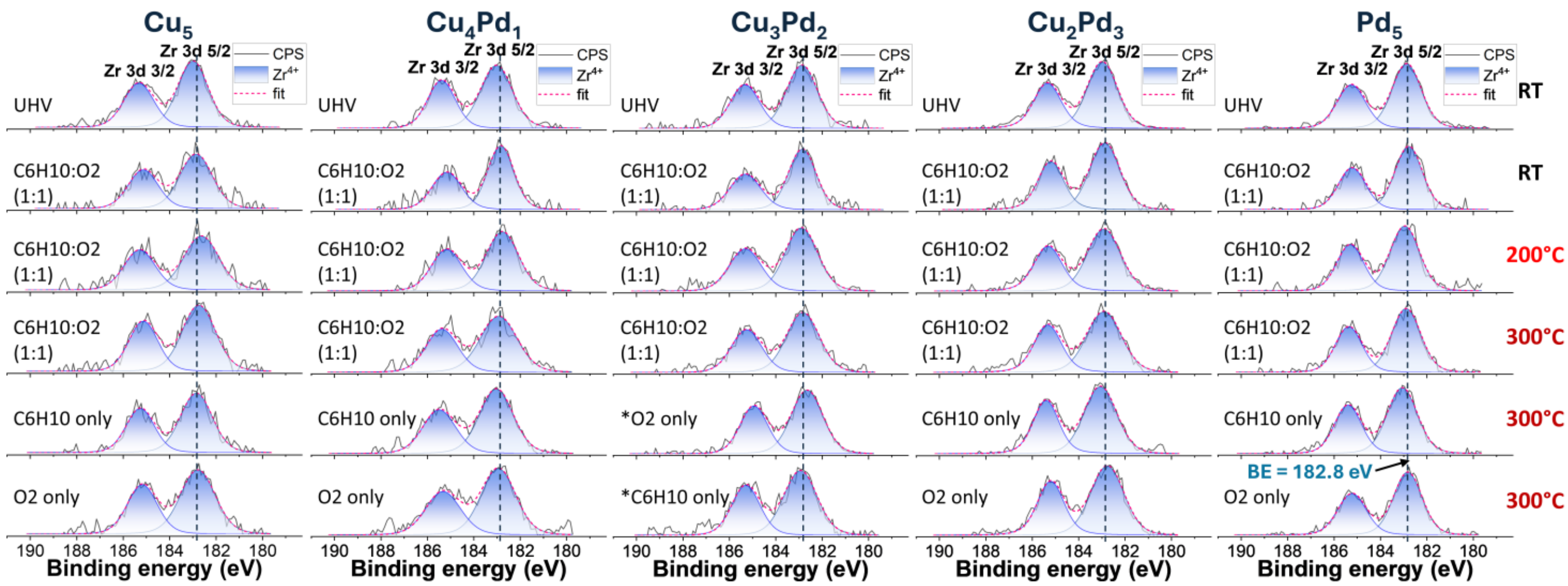

Figure S7. High-resolution Zr 3d spectra for all pentamer catalysts measured by NAP-XPS at different temperatures and under *in situ* gas atmospheres. The accuracy of the determined BE positions is estimated to be ±0.15 eV, reflecting uncertainties associated with both the fitting procedure and the experimental precision of the XPS measurements. All NAP-XPS spectra were normalized to their maximum intensity for better comparison. The Zr $3d_{5/2}$ peak consistently appeared at ~182.8 eV for all samples under all gas exposures and temperatures, indicating that the ultrathin ZrO_2 support remained chemically stable throughout the measurements. No detectable spectral changes can be attributed to the fact that only a small fraction of ZrO_2 sites directly interacts with the clusters, considering their low coverage of 10 %ML.

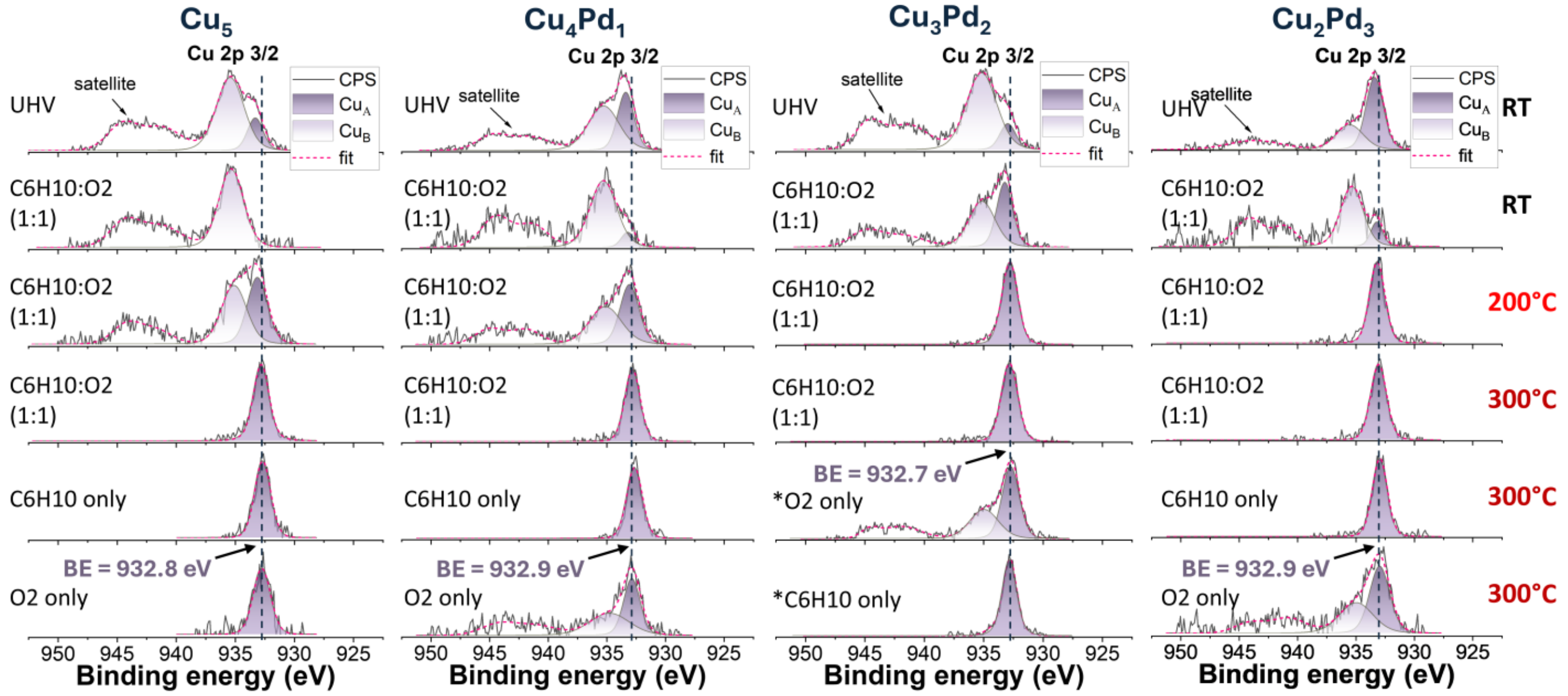

Figure S8. High-resolution Cu $2p_{3/2}$ spectra for all pentamer catalysts measured by NAP-XPS under different temperatures and *in situ* gas atmospheres. The BE positions were referenced to the Si $2p_{3/2}$ core level of Si-Si bond with the BE position of 99.2 eV. The accuracy of the determined BE positions for elemental analysis is estimated to be ±0.15 eV, reflecting uncertainties associated with the fitting procedure and the experimental precision of XPS. All NAP-XPS spectra were normalized to their maximum intensity for better comparison.

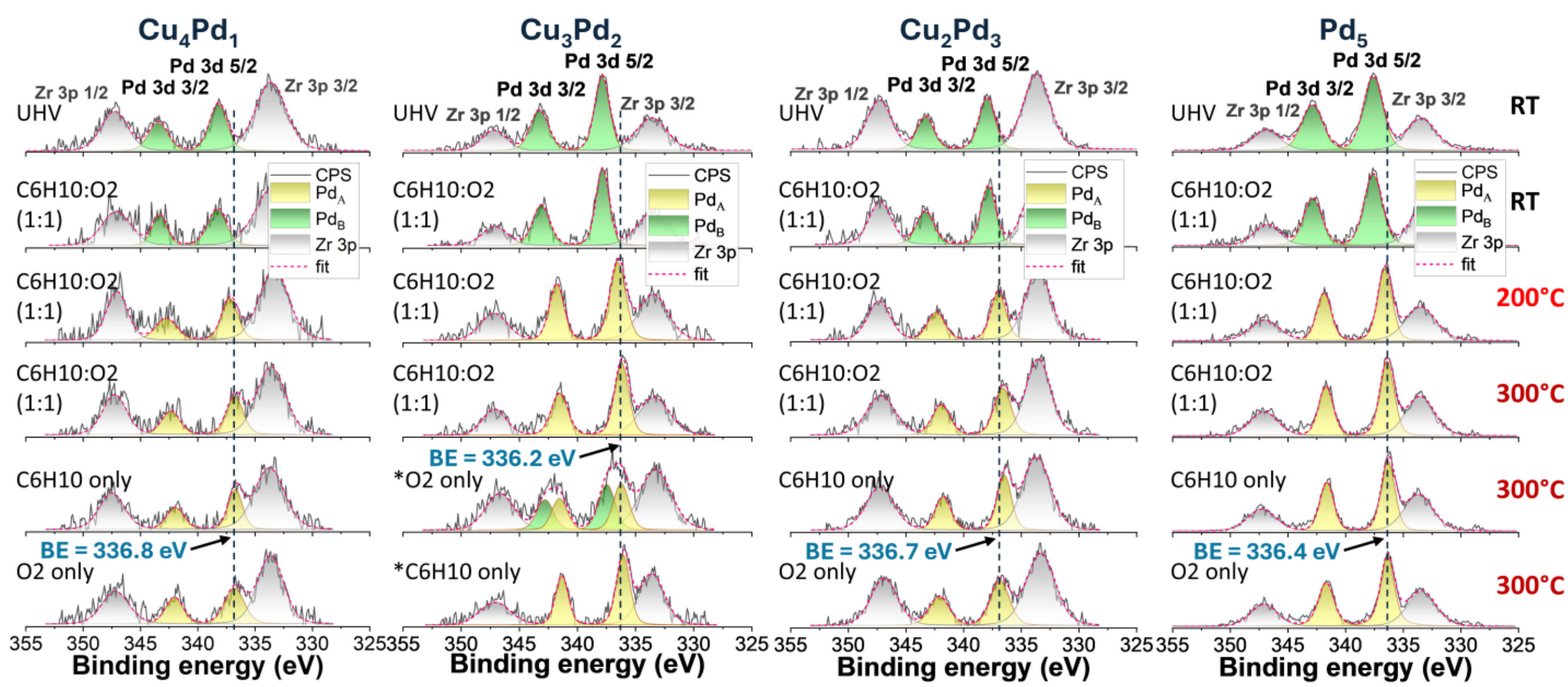

Figure S9. High-resolution Pd 3d spectra for all pentamer catalysts measured by NAP-XPS under different temperatures and *in situ* gas atmospheres. The Pd 3d spectral region partially overlaps with the Zr 3p signal, however, this overlap does not influence the analysis or interpretation of the spectra. The accuracy of the determined BE positions for elemental analysis is estimated to be ±0.15 eV, reflecting uncertainties associated with the fitting procedure and the experimental precision of XPS. All NAP-XPS spectra were normalized to their maximum intensity for better comparison.

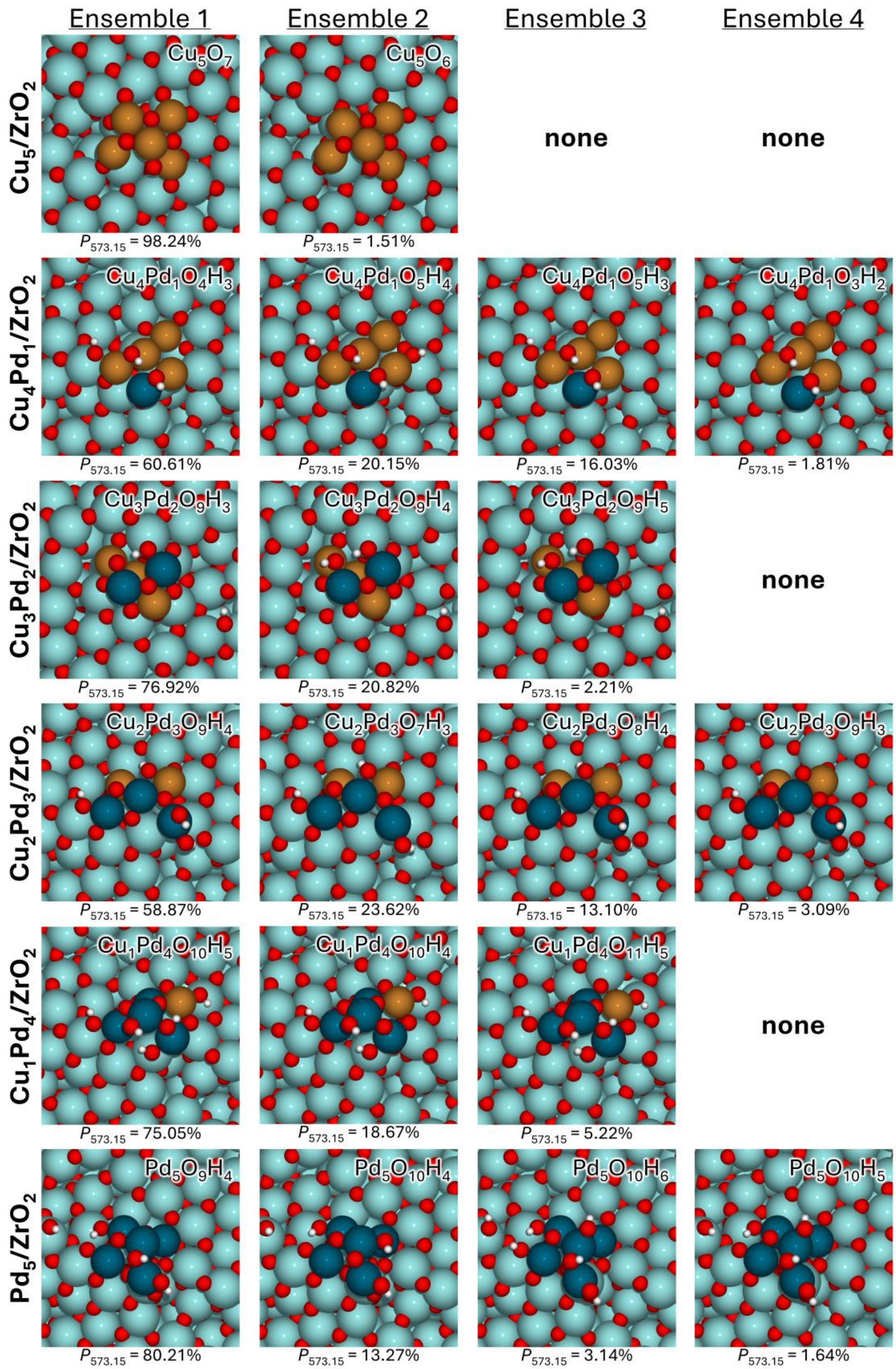

Figure S10. All possible ensemble configurations of Cu_xPd_y pentamers on ZrO_2 under ODH conditions at 300 °C. The Boltzmann population at the given conditions is indicated below each configuration.

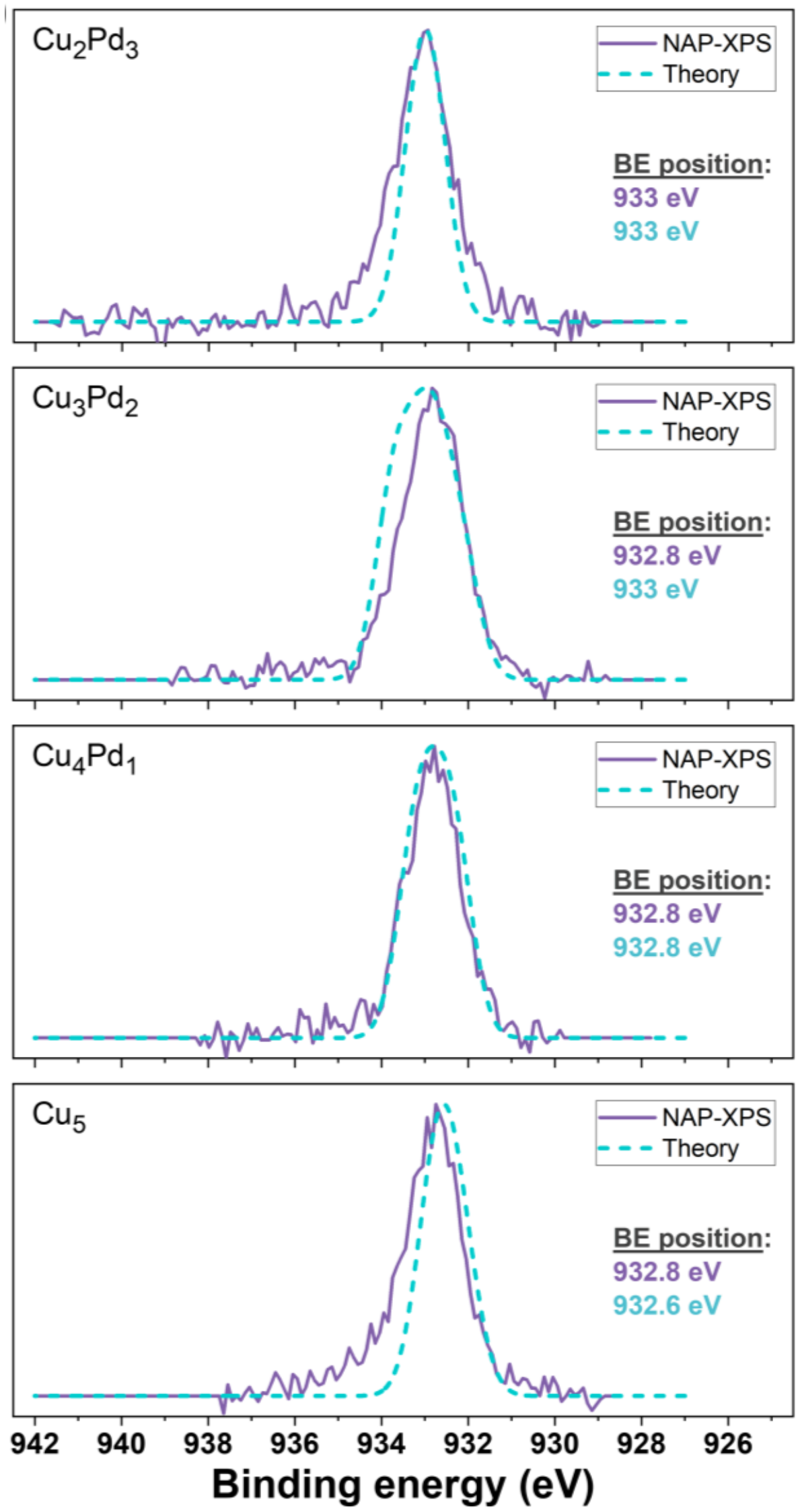

Figure S11. Overlay of experimental NAP-XPS and ensemble-weighted theoretical Cu $2p_{3/2}$ spectra for Cu_2Pd_3, Cu_3Pd_2, Cu_4Pd_1, and Cu_5 obtained/calculated under NAP-XPS reaction conditions at 300 °C (C_6H_{10}:O_2, 1:1 ratio, ~1mbar total pressure). Note: the accuracy of the experimental BE positions is estimated to be ±0.15 eV, reflecting uncertainties associated with the fitting procedure and the experimental precision of XPS.

Section S1. Analysis of the agreement between experimental NAP-XPS and ensemble-weighted theoretical Cu $2p_{3/2}$ spectra (Figure S11).

To connect the ensemble-resolved electronic picture with experimentally accessible spectroscopy, we generated ensemble-weighted theoretical Cu $2p_{3/2}$ spectra for the dominant thermally populated structures sampled under NAP-XPS-matched conditions. Cu $2p_{3/2}$ core-level shifts were calculated using the final-state core-hole approximation by creating a 2p core hole on Cu. Because the calculated quantities are core-level shifts rather than absolute binding energies, the shifts were referenced to an experimental Cu $2p_{3/2}$ energy of 932.6 eV to place the simulated spectra on the experimental binding-energy scale. The core-level contributions were Boltzmann-weighted over the thermally accessible ensembles. The simulated spectra were generated by Gaussian broadening of each contribution using a full width at half maximum of 1.0 eV, followed by normalization of the maximum intensity for comparison with experiment.

Importantly, the NAP-XPS spectra were recorded in a dedicated *in situ* reaction cell that differs from the TPR catalytic reactor used for activity measurements. Therefore, the theoretical XPS calculations were performed using structures sampled under NAP-XPS-matched conditions rather than the TPR-derived reaction environments used for the activity-relevant ensemble and cyclohexene adsorption analysis. During NAP-XPS measurements, the feed consisted of C_6H_{10}:O_2 = 1:1 at a total pressure of ~1 mbar. The gas-phase partial pressures used in the NAP-XPS-matched theoretical sampling are reported in Table S1. This separation is important because subnanometer supported clusters can respond sensitively to oxygen chemical potential, hydroxyl coverage, and the local interfacial environment.[1-3]

Figure S11 overlays the ensemble-weighted theoretical Cu $2p_{3/2}$ spectra with the experimental NAP-XPS data for Cu_2Pd_3, Cu_3Pd_2, Cu_4Pd_1, and Cu_5 at 300 °C. Since the calculated spectra are based on referenced core-level shifts, the comparison focuses on the relative composition-dependent peak shifts rather than on an independent prediction of absolute binding energies. The calculated spectra reproduce the experimental binding-energy range after referencing and capture the main composition-dependent trends, including the higher Cu $2p_{3/2}$ binding energy of Cu_2Pd_3 relative to the more Cu-rich compositions. This agreement should be interpreted as a spectroscopic benchmark of the NAP-XPS-matched structural ensembles, rather than as a one-to-one assignment of formal Cu oxidation states. In particular, the calculated shifts depend on the local O/H coordination, cluster geometry, and final-state screening around each Cu atom.

Remaining differences between the calculated and experimental peak positions and spectral widths are expected because the theoretical spectra are generated from thermodynamically dominant structures on a finite amorphous ZrO_2 model, whereas the experimental spectra average over a broader distribution of cluster adsorption geometries, local support environments, and possible minority Cu-O/Cu-OH configurations. The broader experimental features observed for selected compositions likely reflect this additional structural and chemical heterogeneity. Thus, the comparison in Figure S11 supports the ability of the DFT ensemble model to capture the prevailing Cu core-level response under NAP-XPS conditions, while detailed reproduction of spectral widths and minority components would require more extensive sampling of amorphous ZrO_2 environments and cluster adsorption configurations.

References:

1. Zhai, H.; Alexandrova, A. N., Fluxionality of Catalytic Clusters: When It Matters and How to Address It. *ACS Catal.* **2017,** *7* (3), 1905-1911.
2. Zandkarimi, B.; Alexandrova, A. N., Dynamics of Subnanometer Pt Clusters Can Break the Scaling Relationships in Catalysis. *J. Phys. Chem. Lett.* **2019,** *10* (3), 460-467.
3. Halder, A.; Curtiss, L. A.; Fortunelli, A.; Vajda, S., Perspective: Size selected clusters for catalysis and electrochemistry. *J. Chem. Phys.* **2018,** *148* (11), 110901.

Table S1. Gas-phase partial pressures used for the theoretical calculations under NAP-XPS-matched and TPR-derived environments at 300 °C. For the NAP-XPS entries, the C_6H_{10}:O_2 feed ratio was 1:1 at a total pressure of ~1 mbar. Product partial pressures for the NAP-XPS-matched entries were estimated by applying the cluster-specific conversion and DH/ODH partitioning derived from the 300 °C TPR measurements to the lower NAP-XPS feed pressure. For the TPR entries, the partial pressures correspond to the cluster-specific reaction environments derived from the 300 °C TPR data under a C_6H_{10}:O_2 feed ratio of 1:1 at ~1.1 bar total pressure, with the reactants diluted in He.

Environment	Cluster	C_6H_{10} conversion (%)	p(C_6H_{10}) (mbar)	p(O_2) (mbar)	p(C_6H_6) (mbar)	p(H_2O) (mbar)	p(H_2) (mbar)
NAP-XPS	Cu_5	0.00	0.508	0.508	0.000	0.000	0.000
	Cu_4Pd_1	7.51	0.469	0.498	0.038	0.010	0.044
	Cu_3Pd_2	24.11	0.385	0.466	0.122	0.100	0.117
	Cu_2Pd_3	29.74	0.357	0.449	0.150	0.145	0.135
	Cu_1Pd_4	21.39	0.399	0.486	0.109	0.063	0.177
	Pd_5	49.53	0.256	0.392	0.250	0.290	0.197
TPR	Cu_5	0.00	3.047	3.047	0.000	0.000	0.000
	Cu_4Pd_1	7.51	2.817	2.990	0.229	0.063	0.264
	Cu_3Pd_2	24.11	2.307	2.796	0.735	0.600	0.701
	Cu_2Pd_3	29.74	2.141	2.694	0.900	0.869	0.812
	Cu_1Pd_4	21.39	2.395	2.918	0.652	0.376	1.062
	Pd_5	49.53	1.538	2.352	1.502	1.740	1.185

Section S2. Free-energy calculations for the first O-assisted C-H activation.

Reaction-path calculations: The initial states were selected from the two most populated oxidized configurations of each active composition under the TPR-derived reaction environment: $Cu_2Pd_3(O_9H_4$, 58.9%) and $Cu_2Pd_3(O_7H_3$, 23.6%); $Pd_5(O_9H_4$, 80.2%) and $Pd_5(O_{10}H_4$, 13.3%); and $Cu_3Pd_2(O_9H_3$, 76.9%) and $Cu_3Pd_2(O_9H_4$, 20.8%). Cyclohexene adsorption modes containing a short, poised C-H···O contact to cluster-bound O were used for the first H-transfer calculations.

To improve reliability in the bond-breaking region and mitigate potential MLIP softening, the training set was augmented with 121 additional DFT-labeled configurations sampled from preliminary reaction pathways, including the bridging C-H···O transfer region. A single shared reaction-path-refined MACE model was used for all six pathways. The NEB bands were adaptively densified, and the H-transfer region was refined with climbing-image NEB. Each transition state was confirmed as a first-order saddle by a single imaginary mode in the adsorbate partial Hessian (170-1015 cm^{-1}). Electronic and free-energy barriers are referenced to the poised adsorbed reactant.

Free-energy treatment: Harmonic vibrational free energies of the adsorbed initial, transition, and final states were evaluated at 573.15 K using a partial Hessian for the 16-atom cyclohexene adsorbate, with the supported cluster treated as a rigid bath. The finite-difference displacement was 0.01 Å; low-frequency modes were floored at 12 cm^{-1}, and the single reaction-coordinate imaginary mode was excluded from the transition-state free energy. Gas-phase c-C_6H_{10} was treated with the ideal-gas approximation at 573.15 K and 1 bar, including translational, rotational, and vibrational contributions for a nonlinear molecule with symmetry number 1 and spin 0.

The adsorption free energy was defined as $\Delta G_{\text{ads}}(573.15\text{K}) = G_{\text{adsorbed}} - E_{\text{bare}} - G_{\text{gas}}(573.15\text{K})$, where E_{bare} is the electronic energy of the bare supported cluster in the rigid-bath approximation. For comparison of the sampled first-step pathways, the rate contribution would scale as $r \propto \sum_i P_{\text{cluster},i} P_{\text{mode},i} \exp\left(-\Delta G_i^{\ddagger}/k_B T\right)$; Because adsorption-mode populations were not evaluated, this expression is not used to identify a kinetically dominant or operative pathway. Instead, one representative pathway per composition was selected from a thermally populated oxidized cluster configuration combining a low intrinsic barrier with a non-negligible cluster population. This designation applies only to the first H-transfer step and does not assign the global rate-determining step or experimental activity ranking.

Results: In all six pathways, H transfers from c-C_6H_{10} to cluster-bound O through a bridging C-H···O transition structure. The adsorbed-state $\Delta G^{\ddagger}$ values are 0.12 and 0.34 eV for Cu_2Pd_3, 0.34 and 0.45 eV for Pd_5, and 0.36 and 1.09 eV for Cu_3Pd_2. Five configurations have positive ΔG_{ads} values of +0.30 to +0.46 eV, indicating low but finite equilibrium coverage, whereas $Cu_2Pd_3(O_7H_3)$ has ΔG_{ads} = -0.16 eV. Across all six configurations, the gas-referenced effective barrier $\Delta G_{ads} + \Delta G^{\ddagger}$ ranges from 0.18 to 1.39 eV; the values for the selected representative pathways are 0.45, 0.73, and 0.82 eV for Cu_2Pd_3, Pd_5, and Cu_3Pd_2, respectively. The results establish an accessible first O-assisted H-transfer pathway and strong composition- and O-site dependence but do not determine the global rate-determining step or H_2/H_2O branching.

The final pathways are reported at the MLIP level. For this soft, weakly adsorbed system, an approximate 0.1-0.2 eV uncertainty is assigned, and the harmonic treatment of low-frequency adsorbate modes is an additional limitation. Accordingly, the common O-assisted mechanism and configuration-dependent trends are emphasized over fine numerical differences among the low barriers.

Table S2. Electronic and free-energy results for the six first O-assisted C-H activation pathways at 573.15 K. $\Delta E^{\ddagger}$ and $\Delta G^{\ddagger}$ are referenced to the poised adsorbed reactant; ΔG_{ads} is referenced to gas-phase c-C_6H_{10} at 1 bar plus the bare supported cluster; $\Delta G_{ads} + \Delta G^{\ddagger}$ is the gas-referenced effective barrier. Energies are in eV, and $|\nu^{\ddagger}|$ is the magnitude of the single imaginary transition-state frequency in cm^{-1}.

Composition / configuration	P_{573K} (%)	$\Delta E^{\ddagger}$	$\Delta G^{\ddagger}$	ΔE_{rxn}	ΔG_{rxn}	ΔE_{ads}	ΔG_{ads}	$\Delta G_{ads}+\Delta G^{\ddagger}$	$\|\nu^{\ddagger}\|$
Cu_2Pd_3 (O_9H_4)	58.9	0.08	0.12	-0.71	-0.68	-0.51	+0.33	0.45	345
Cu_2Pd_3 (O_7H_3)	23.6	0.35	0.34	-0.56	-0.49	-1.31	-0.16	0.18	698
Pd_5 ($O_{10}H_4$)	13.3	0.20	0.34	-1.84	-1.42	-0.40	+0.39	0.73	170
Pd_5 (O_9H_4)	80.2	0.44	0.45	-0.12	-0.06	-0.56	+0.41	0.86	1015
Cu_3Pd_2 (O_9H_3)	76.9	0.30	0.36	+0.08	+0.09	-0.20	+0.46	0.82	978
Cu_3Pd_2 (O_9H_4)	20.8	1.13	1.09	+0.24	+0.55	-0.56	+0.30	1.39	352

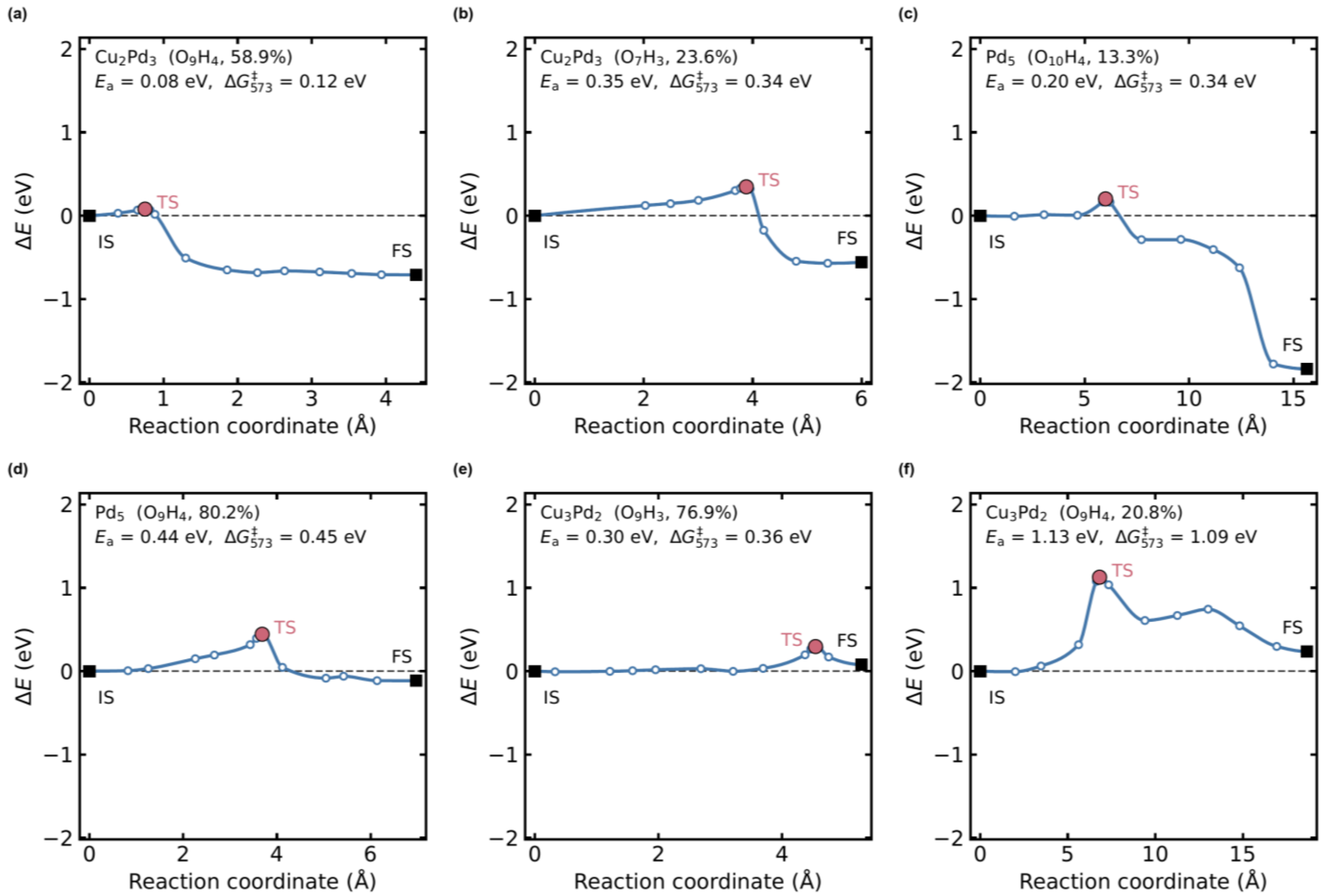

Figure S12. MLIP-level NEB profiles for the first O-assisted H transfer from c-C_6H_{10} to cluster-bound O on the two populated oxidized configurations examined for each composition: (a) Cu_2Pd_3(O_9H_4, 58.9%), (b) Cu_2Pd_3(O_7H_3, 23.6%), (c) Pd_5($O_{10}H_4$, 13.3%), (d) Pd_5(O_9H_4, 80.2%), (e) Cu_3Pd_2(O_9H_3, 76.9%), and (f) Cu_3Pd_2(O_9H_4, 20.8%). The electronic barriers ($\Delta E^‡$) are 0.08, 0.35, 0.20, 0.44, 0.30, and 1.13 eV, respectively, and the corresponding $\Delta G^‡$ values at 573.15 K are 0.12, 0.34, 0.34, 0.45, 0.36, and 1.09 eV. Energies are referenced to the poised adsorbed reactant; each transition state is a bridging C-H···O saddle confirmed by a single imaginary mode.
